# Chewing Lice From the Wings of Migrating Shorebirds: Diversity, Host Specificity, and Associations With Host Body Condition

**DOI:** 10.1002/ece3.74013

**Published:** 2026-07-25

**Authors:** Maciej Bartos, Radosław Włodarczyk, Amelia Paszkowska, Tomasz Rewicz, Maciej Kamiński, Jan Rapczyński, Patryk Fiutek, Piotr Minias

**Affiliations:** ^1^ Department of Biodiversity Studies and Bioeducation, Faculty of Biology and Environmental Protection University of Lodz Lodz Poland; ^2^ Faculty of Biology and Environmental Protection, Institute of Biophysics University of Lodz Lodz Poland; ^3^ Department of Invertebrate Zoology and Hydrobiology, Faculty of Biology and Environmental Protection University of Lodz Lodz Poland; ^4^ Forestry Student Scientific Association, Ornithological Section Warsaw University of Life Sciences Warszawa Poland; ^5^ Independent Researcher Przybynow Poland

**Keywords:** bird parasites, chewing lice, DNA barcoding, host condition, migration strategy, Phthiraptera

## Abstract

Ectoparasites may negatively affect their hosts throughout life, especially during breeding and other energetically demanding periods. In birds, feather‐feeding ectoparasites may reduce host condition and performance during seasonal migrations. This study aimed to investigate the diversity of wing‐collected chewing lice (Phthiraptera) and their associations with two groups of avian hosts, Charadriidae and Scolopacidae. We examined 1403 individuals from 20 migratory shorebird species to estimate louse prevalence and abundance, and a subsample of lice was identified using microscopic examination and DNA barcoding. In total, 26 lice species were identified across all hosts. Measures of louse prevalence and abundance varied widely among host species (9%–94% infested individuals and 0.2–26.4 lice per individual). Infestation differed significantly between age classes (higher in first‐year than in adult birds) and between habitats used by birds during migration (higher at an artificial reservoir than in a natural river valley). Notably, louse abundance was negatively associated with condition (fat load and size‐corrected body mass) in a time‐constrained long‐distance migrant, the wood sandpiper *Tringa glareola* , whereas no such pattern was found in a short‐distance migrant with fewer time constraints during migration, the common snipe *Gallinago gallinago* . Our findings suggest that chewing lice may negatively influence avian hosts during critical stages of the annual cycle, such as migration, but these effects may be modulated by host behavior, particularly migration strategy.

## Graphical abstract

We investigated the diversity, prevalence, and abundance of chewing lice in 1403 individuals representing 20 migratory shorebird species from the families Charadriidae and Scolopacidae, identifying 26 louse species through microscopy and DNA barcoding. Louse infestation varied markedly among host species and was higher in first‐year birds than in adults, as well as at an artificial reservoir compared with a natural river valley. Louse abundance was negatively associated with body condition in the time‐constrained long‐distance migrant *Tringa glareola* , but not in the short‐distance migrant *Gallinago gallinago* , suggesting that the effects of ectoparasites during migration may depend on host migration strategy.

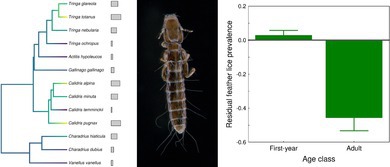

## 1 Introduction

Parasites play a key role in shaping the ecology and evolution of their hosts (Zuk and Loye 1991; Clayton and Moore 1997). Although the remarkable diversity of avian parasites has long been recognized (Rothschild and Clay 1952), research on parasites in wild birds accelerated markedly following the seminal work of Hamilton and Zuk (1982), which proposed that elaborate male sexual traits function as honest signals of parasite resistance under parasite‐mediated sexual selection. Since then, our understanding of avian parasite diversity and their impacts on hosts has expanded substantially (Møller and Saino 1994; Proctor and Owens 2000; Dunn et al. 2021). However, much of the research on host–parasite interactions has focused on groups that strongly affect bird condition and survival, particularly hematophagous arthropods (Clayton and Moore 1997; Proctor and Owens 2000; Pryor and Casto 2015; Nogueira et al. 2023). Although these parasites interact with birds only intermittently, they may impose strong direct costs on hosts and, additionally, act as vectors of avian haemosporidian parasites such as *Plasmodium*, *Haemoproteus*, and *Leucocytozoon* (Santiago‐Alarcon et al. 2012). In contrast, chewing lice (Phthiraptera) are permanent host‐associated arthropods that spend their entire life cycle on the host and remain in direct contact with feathers and skin. Although recent work has substantially advanced our understanding of chewing‐louse life history, feeding biology, off‐host survivorship, morphological diversification, and genomic evolution, many aspects of their diversity and host associations remain incompletely resolved (Galloway and Lamb 2021; Johnson 2022; Kolenčík et al. 2025; Gustafsson et al. 2024).

Chewing lice are a widespread clade of insects (Insecta: Psocodea) that comprise obligatory ectoparasites of birds and mammals. They primarily feed on feathers and dermal debris, although some species also consume blood (Johnson and Clayton 2003). More than 4500 species of chewing lice are currently recognized worldwide, with most of them (approximately 80%) parasitizing birds (Price et al. 2003) and occurring in nearly all extant avian families (173 out of 189) (Smith et al. 2011). Their biology is tightly linked to their hosts and their ability to survive off the host varies among lineages, with amblyceran lice generally surviving for shorter periods than ischnoceran lice, especially when host feathers are available as food (Tompkins and Clayton 1999; Clayton and Johnson 2003; Kolenčík et al. 2025). Dispersal occurs when two hosts come into direct and prolonged contact, for example between parents and offspring (vertical transmission). However, transmission can also take place during brief contact lasting only a few seconds, as occurs during mating (horizontal transmission) (Clayton and Tompkins 1994; Hillgarth 1996; Bush and Clayton 2006). Horizontal transmission may further occur when hosts of the same or different species share nesting sites (Johnson et al. 2002; Weckstein 2004; Banks et al. 2006), aggregate to feed or roost, form flocks (de Brooke and Nakamura 1998), or use common dust baths (Hoyle 1938; Clay 1949). Some lice are also capable of dispersing via phoresy by using hippoboscid flies (Harbison et al. 2009) or snipe flies (Romiti et al. 2026) as vectors. Infestation intensity varies among hosts, but severe infestations can negatively influence mating success (Clayton 1990; Moreno‐Rueda and Hoi 2012) and survival (Brown et al. 1995; Clayton et al. 1999). By damaging feathers, lice can impair thermal insulation (Booth et al. 1993) and flight performance (Barbosa et al. 2002). Chewing lice belonging to the clade Amblycera frequently damage the host skin, which may result in blood loss, inflammation, and superficial skin lesions or scab formation (Dik 2006; Prelezov et al. 2006; Clayton et al. 2008). In addition, some lice can act as vectors or intermediate hosts for other parasites, including filarial nematodes such as *Eulimdana* spp. and *Sarconema eurycerca* (Cohen et al. 1991; Bartlett 1993). Infested birds often invest considerable time in preening, which suggests that controlling louse populations may be essential for maintaining good health (Cotgreave and Clayton 1994; Clayton et al. 2005, 2010).

Chewing louse prevalence, abundance, and species richness are shaped by a wide range of abiotic and biotic factors. Lice are permanent ectoparasites, which means that the host body serves as their sole habitat, often conceptualized as a “habitat island” (Rózsa 1997), where they occupy and adapt to distinct ecological niches. Chewing lice exhibit a high degree of specialization not only toward their hosts, but also toward particular regions of the host body they occupy. Many species specialize in particular body regions, such as the wings, head, or torso, whereas others are more generalist in their microhabitat use (Bush and Clayton 2006; Johnson et al. 2012; Sweet and Johnson 2018; Johnson and Doña 2024). The environment within the plumage of a homeothermic bird is not constant and may be modified by various external factors (Merino and Potti 1996; Møller 2010). In addition, surface temperatures differ among host body regions, contributing to microclimatic heterogeneity within the plumage (Kolenčík et al. 2025). Consequently, environmental variables such as ambient temperature and humidity can influence louse populations (Janovy et al. 1997; Moyer, Gardiner, and Clayton 2002; Møller 2010). Low humidity is often associated with reduced louse prevalence and abundance (Moyer, Drown, and Clayton 2002), although this pattern may differ among louse species occurring on the same host (Bush et al. 2009), and some studies have reported high louse abundance even in arid environments (Carrillo et al. 2007). Ambient temperature also appears to have context‐dependent effects across studies. Louse prevalence and species richness in the domestic pigeon *Columba livia domestica* were higher during the warmest season (da Cunha Amaral et al. 2017), whereas Sychra et al. (2011) found that birds forming flocks under winter conditions may show increased louse prevalence. These findings suggest that the diversity of chewing lice occupying different ecological niches on the same host may lead to distinct responses to the same abiotic factors. Biotic factors, including host body mass, sex, age, bill shape and length, flocking behavior, feeding ecology, and migratory behavior, have also been shown to influence louse populations (Poulin 1997; Dik et al. 2010, 2015, 2017; Galloway and Lamb 2017; Durkin et al. 2015; Chu et al. 2019; Lamb and Galloway 2019; Brewer and Sweet 2023). However, reported relationships are often context‐dependent, varying among host–parasite systems, ecological settings, spatial and temporal scales. Consequently, the extent to which ecological factors shape louse prevalence and abundance remains debated (Moyer, Drown, and Clayton 2002; Sychra et al. 2011; da Cunha Amaral et al. 2017; Sajid and Ehsan 2017; Bush and Clayton 2018; Chu et al. 2019; Galloway and Lamb 2021).

Although the global avian chewing‐louse fauna has been compiled in broad taxonomic checklists and host‐association summaries (Price et al. 2003; Rózsa and Vas 2015), substantial gaps remain in species‐level diversity and in the delimitation of species, including undescribed taxa, cryptic lineages, and insufficiently revised genera (Valim and Weckstein 2013; Escalante et al. 2016; Kolenčík et al. 2024; Gustafsson et al. 2025). Moreover, knowledge of wild bird‐louse associations remains uneven: while several coevolutionary adaptations are well documented, taxonomic and geographic coverage of host‐louse associations remains incomplete, and the ecological consequences of infestation in natural populations are still poorly understood (Johnson et al. 2005, 2012; Bush et al. 2010; Brewer and Sweet 2023). Among avian orders, chewing lice of Charadriiformes appear to be comparatively well studied relative to those of many other bird groups (Clay 1959, 1962; Złotorzycka et al. 1997; Hughes and Page 2007; Gustafsson and Olsson 2012a, 2012b; Gustafsson et al. 2019; Grossi et al. 2023). Nevertheless, knowledge of host‐louse associations within this order remains geographically uneven. Recent works have substantially improved the state of the art for some regions and at broader scales (e.g., Grossi et al. 2023; Dik et al. 2023), but in Europe much of the available information still comes from scattered country‐level reports and outdated host–louse records (reviewed in Grossi et al. 2023). In Poland, published data on chewing lice of Charadriiformes derive largely from studies conducted several decades ago (Złotorzycka 1967, 1976, 1978; Złotorzycka and Modrzejewska 1988; Złotorzycka et al. 1997). Moreover, the most comprehensive study of lice from Charadriidae and Scolopacidae in Poland (Złotorzycka et al. 1997) was based on material from only 11 species, representing only a limited subset of the more than 60 shorebird species that regularly migrate through or occur as vagrants in Poland (Tomiałojć and Stawarczyk 2003).

Although chewing lice from wild birds are increasingly studied, documenting their diversity remains challenging because of their high species richness, the difficulty of accurate identification, and the limited taxonomic capacity available for many groups (Kolenčík et al. 2024; Gustafsson et al. 2025). Molecular tools, particularly DNA barcoding of the mitochondrial cytochrome c oxidase subunit I (COI) gene, can help verify uncertain identifications and reveal cryptic diversity, but their effectiveness depends on well‐curated reference libraries linked to reliably identified voucher specimens (Hebert et al. 2003; Rimet et al. 2021). However, the reference library for chewing lice remains far from complete (Grossi et al. 2014; Lee et al. 2022). According to the Barcode of Life Data Systems (BOLD; http://v4.boldsystems.org; Ratnasingham and Hebert 2007) database, public barcodes are available for only 78 species of Philopteridae and 100 species of Ancistronidae, representing ~5% of the ~3500 described species and highlighting the need for additional sequences linked to reliably identified voucher specimens. These limitations motivated us to complement morphology‐based identifications with molecular data.

In the present study, we aimed to quantify the diversity, host specificity, and associations with host body condition in chewing lice collected from the wings of Charadriidae and Scolopacidae shorebirds migrating through Poland. To achieve this, we collected data on relative louse prevalence and abundance in approximately 1400 individuals representing 20 shorebird species and examined factors responsible for variation in infestation rates. Specifically, we expected that both louse prevalence and abundance may show pronounced variation among host species, host age classes (higher in young individuals e.g., due to limited preening efficiency or naïve immune responses) and ecological contexts (stopover habitat type and migration phenology). We also collected nearly 250 louse specimens for molecular (barcoding) and morphological identification. Furthermore, we sought to assess the potential impact of chewing lice collected from the wings on host condition. This part of the study focused on two shorebird species with contrasting migration strategies: the short‐distance migrant, the common snipe *Gallinago gallinago* , and the long‐distance migrant, the wood sandpiper *Tringa glareola* . We hypothesized that associations of louse infestation with host body condition may differ between the two focal host species because of their contrasting migration strategies. Particularly, we predicted that the negative effects of lice on host condition may be more pronounced in a long‐distance migrant (wood sandpiper) that is more energetically constrained during migration.

## 2 Materials and Methods

### 2.1 Study Site and Fieldwork

Samples were collected during autumn shorebird migration from mid‐July to late September in 2019–2021. Birds were captured at two inland stopover sites in central Poland: the artificial Jeziorsko Reservoir on the Warta River (51°47′ N, 18°40′ E) and the natural Middle Vistula River valley (Rembeza's Island; 51°57′ N, 21°16′ E). The Jeziorsko site consisted of extensive mudflats rich in benthic invertebrates, whereas the Middle Vistula site was characterized by sandy islets, muddy riverbanks, and locally shallow riverbed sections, all of which provided favorable foraging conditions for migrating shorebirds. Birds were trapped using walk‐in traps and individually marked with metal rings placed on the left tarsus. The age of each captured bird was determined as either first‐year or adult. Age determination was based on plumage characteristics (including coloration and patterning of the wing coverts; Baker 2016) and feather wear (in autumn first‐year shorebirds have fresh plumage, whereas adult individuals show visibly worn feathers, especially primaries; Demongin 2016). For each individual, we collected standard morphometric measurements, including body mass (measured to the nearest 1 g using an electronic scale) and wing length (measured to the nearest 1 mm using a stopped ruler). The amount of visible subcutaneous fat in the furcular and axillary regions (hereafter referred to as fat load) was assessed using a special scale developed for shorebirds: a score of zero indicates the absence of visible subcutaneous fat in the axillary region and furculum, while a score of four reflects deeply concave fat deposit in the furcular region and all musculature covered by a thick fat layer in the axillary region (Meissner 2000). Fat load together with body mass corrected for structural size (see Statistical analyses) served as indices of body condition, i.e., the measures of energy reserves in migrating birds.

Immediately after ringing procedures, birds were visually inspected for the presence of lice. To do this, we carefully examined the upper and underwing feathers on both wings, including all remiges, coverts, scapulars, and axillaries, manually parting or gently blowing along the full length of each rachis. Because only contour feathers occur on the wings of shorebirds (with no down feathers), it is possible to effectively inspect the entire wing, including the skin surface. Such inspection was technically unfeasible for other parts of the host body, which are largely covered by a thick layer of down and contour feathers. For this reason, our assessment was limited to lice present on the wings at the time of examination; individuals located elsewhere on the body were not recorded. No supplementary magnification was used during inspections. All data on louse infestation were collected by five authors (MK, PM, RW, JR, and PF) following a standardized protocol in which the same parts of the wing were examined in a consistent order (beginning with large flight feathers, continuing with smaller wing coverts, and ending with inspection of the skin surface). Each wing was inspected for 1 min under good natural light conditions. For each host we quantified the presence of lice on the wings as a binary variable (present or absent) and recorded abundance as the total number of lice counted (adults and nymphs jointly). This visual inspection protocol was designed to be compatible with standard ringing procedures and to allow rapid field assessment of louse infestation without prolonged handling or the use of chemical agents. As such, it enabled broad screening of birds under routine field conditions while minimizing additional disturbance associated with parasite sampling.

While we acknowledge that our simplified protocol may not yield an accurate estimate of the true abundance of lice on the wings, our aim was to quantify relative differences in louse infestation among individuals and species rather than to determine the absolute number of lice on each bird. For this reason, we required the protocol to provide repeatable rather than strictly accurate estimates. To assess this, we initially used 20 hosts (shorebirds) to quantify measurement repeatability. After the first louse count, each bird was placed back into a container for 10 min, after which the louse count was repeated either by the same or a different observer. Repeatability of lice abundance was high and statistically significant (*R* = 0.86, 95% CI: 0.682–0.944, *p* < 0.001), as calculated using the *irr* package (Gamer et al. 2012) in the R statistical environment (R Development Core Team, Vienna, Austria). These results indicated that our standardized protocol provided a reliable (i.e., repeatable) method for quantifying relative differences in louse prevalence and abundance among individuals and species. In practice, the method effectively captured interindividual variation in infestation levels by lice on the wings, as our abundance estimates spanned two orders of magnitude (see Results for details).

We used three proxies to quantify interspecific variation in louse infestation: (i) relative prevalence, defined as the proportion of infested hosts within each species; (ii) relative abundance, defined as the average number of counted lice across all hosts, including both infested and noninfested individuals, within each species; and (iii) relative intensity, defined as the average number of counted lice across infested hosts within each species. In total, louse infestation data were collected from 1403 host individuals (364–639 per year) representing 20 Charadriidae and Scolopacidae species belonging to eleven genera and six subfamilies (Table 1). Infestation parameters were quantified and reported for 13 species with at least five individuals inspected (for prevalence and abundance) or at least five individuals infested (for intensity) (Table 2).

**TABLE 1:** Species‐level records of chewing lice collected from shorebirds during autumn migration through central Poland.

Bird family	Bird subfamily	Bird species	No. host individuals with lice identified to species (*N*)	Louse species	Host individuals with the louse species recorded, *n*/*N* (%)
Charadriidae	Pluvialinae	*Pluvialis squatarola*	1	*Actornithophilus flavipes*	1/1 (100)
	Charadriinae	*Vanellus vanellus*	0	—	—
		*Charadrius dubius*	6	*Actornithophilus ochraceus*	5/6 (83.3)
				*Quadraceps bicuspis*	1/6 (16.7)
		*Charadrius hiaticula*	12	*Actornithophilus ochraceus*	9/12 (75.0)
				*Quadraceps hiaticulae*	4/12 (33.3)
Scolopacidae	Numeniinae	*Numenius arquata*	2	*Lunaceps numenii*	2/2 (100)
				*Cummingsiella ovalis*	1/2 (50)
	Arenariinae	*Arenaria interpres*	1	*Actornithophilus bicolor*	1/1 (100)
				*Quadraceps strepsilaris*	1/1 (100)
		*Calidris ferruginea*	2	*Lunaceps falcinellus*	2/2 (100)
				*Lunaceps holophaeus*	2/2 (100)
		*Calidris pugnax*	11	*Actornithophilus pustulosus*	5/11 (45.5)
				*Lunaceps holophaeus*	7/11 (63.6)
		*Calidris temminckii*	2	*Lunaceps falcinellus*	2/2 (100)
		*Calidris minuta*	3	*Lunaceps falcinellus*	3/3 (100)
		*Calidris alpina*	43	*Lunaceps schismatus*	42/43 (97.7)
				*Carduiceps meinertzhageni*	1/43 (2.3)
	Scolopacinae	*Gallinago gallinago*	34	*Rhynonirmus scolopacis*	33/34 (97.1)
				*Austromenopon durisetosum*	1/34 (2.9)
		*Lymnocryptes minimus*	0	—	—
	Tringinae	*Xenus cinereus*	0	—	—
		*Actitis hypoleucos*	30	*Quadraceps ravus*	24/30 (80.0)
				*Actornithophilus flumineus*	4/30 (13.3)
				*Austromenopon hystriculum*	1/30 (3.3)
				*Quadraceps hiaticulae*	1/30 (3.3)
				*Saemundssonia platygaster frater*	1/30 (3.3)
		*Tringa ochropus*	1	*Quadraceps ochropi*	1/1 (100)
		*Tringa erythropus*	3	*Quadraceps furvus*	3/3 (100)
		*Tringa nebularia*	7	*Quadraceps similis*	5/7 (71.4)
				*Actornithophilus paludosus*	4/7 (57.1)
		*Tringa totanus*	5	*Quadraceps obtusus*	5/5 (100)
				*Actornithophilus totani*	2/5 (40.0)
		*Tringa glareola*	73	*Quadraceps obscurus*	72/73 (98.6)
				*Lunaceps falcinellus*	1/73 (1.4)

*Note:* For each host species, *N* denotes the number of host individuals from which at least one louse specimen was identified to species, and *n* denotes the number of host individuals in which a given louse species was recorded. Because a single host individual could harbor more than one louse species, percentages for different louse species within a host species do not necessarily sum to 100. Species‐level identifications were obtained only for a subset of inspected birds; therefore, these data should be interpreted as conservative records of wing‐collected lice rather than a complete estimate of total louse diversity on each host species.

**TABLE 2:** Shorebird species (*N* = 20) inspected for the occurrence of chewing lice during autumn migration through central Poland.

Bird family	Bird subfamily	Bird species	No. individuals	Prevalence (mean ± SE)	Abundance (mean ± SE)	Intensity (mean ± SE)
Charadriidae	Pluvialinae	*Pluvialis squatarola*	1	NA	NA	NA
	Charadriinae	*Vanellus vanellus*	5	0.20 ± 0.20	0.2 ± 0.2	NA
		*Charadrius dubius*	53	0.21 ± 0.06	0.6 ± 0.3	2.8 ± 0.8
		*Charadrius hiaticula*	36	0.58 ± 0.08	3.8 ± 1.6	6.6 ± 2.6
Scolopacidae	Numeniinae	*Numenius arquata*	3	NA	NA	NA
	Arenariinae	*Arenaria interpres*	2	NA	NA	NA
		*Calidris ferruginea*	3	NA	NA	NA
		*Calidris pugnax*	26	0.92 ± 0.05	26.4 ± 4.0	28.6 ± 3.6
		*Calidris temminckii*	11	0.09 ± 0.09	0.8 ± 0.8	NA
		*Calidris minuta*	8	0.63 ± 0.18	4.5 ± 2.4	7.2 ± 3.4
		*Calidris alpina*	87	0.85 ± 0.04	6.8 ± 0.7	8.0 ± 0.7
	Scolopacinae	*Gallinago gallinago*	417	0.29 ± 0.02	0.8 ± 0.1	2.8 ± 0.4
		*Lymnocryptes minimus*	1	NA	NA	NA
	Tringinae	*Xenus cinereus*	1	NA	NA	NA
		*Actitis hypoleucos*	199	0.15 ± 0.03	0.3 ± 0.1	2.1 ± 0.3
		*Tringa ochropus*	8	0.13 ± 0.13	0.4 ± 0.4	NA
		*Tringa erythropus*	3	NA	NA	NA
		*Tringa nebularia*	15	0.53 ± 0.13	10.1 ± 4.0	18.9 ± 5.9
		*Tringa totanus*	18	0.94 ± 0.06	8.6 ± 1.9	9.1 ± 1.9
		*Tringa glareola*	506	0.65 ± 0.02	5.7 ± 0.5	8.8 ± 0.7

*Note:* Louse infestation parameters were quantified for species with sample sizes of at least five individuals inspected (for prevalence and abundance) or at least five individuals infested (for intensity). All parameters were measured using a simplified protocol designed to capture variation in infestation among host individuals and species, although the method may underestimate true infestation levels.

### 2.2 Louse Species Identification

**FIGURE 1:**
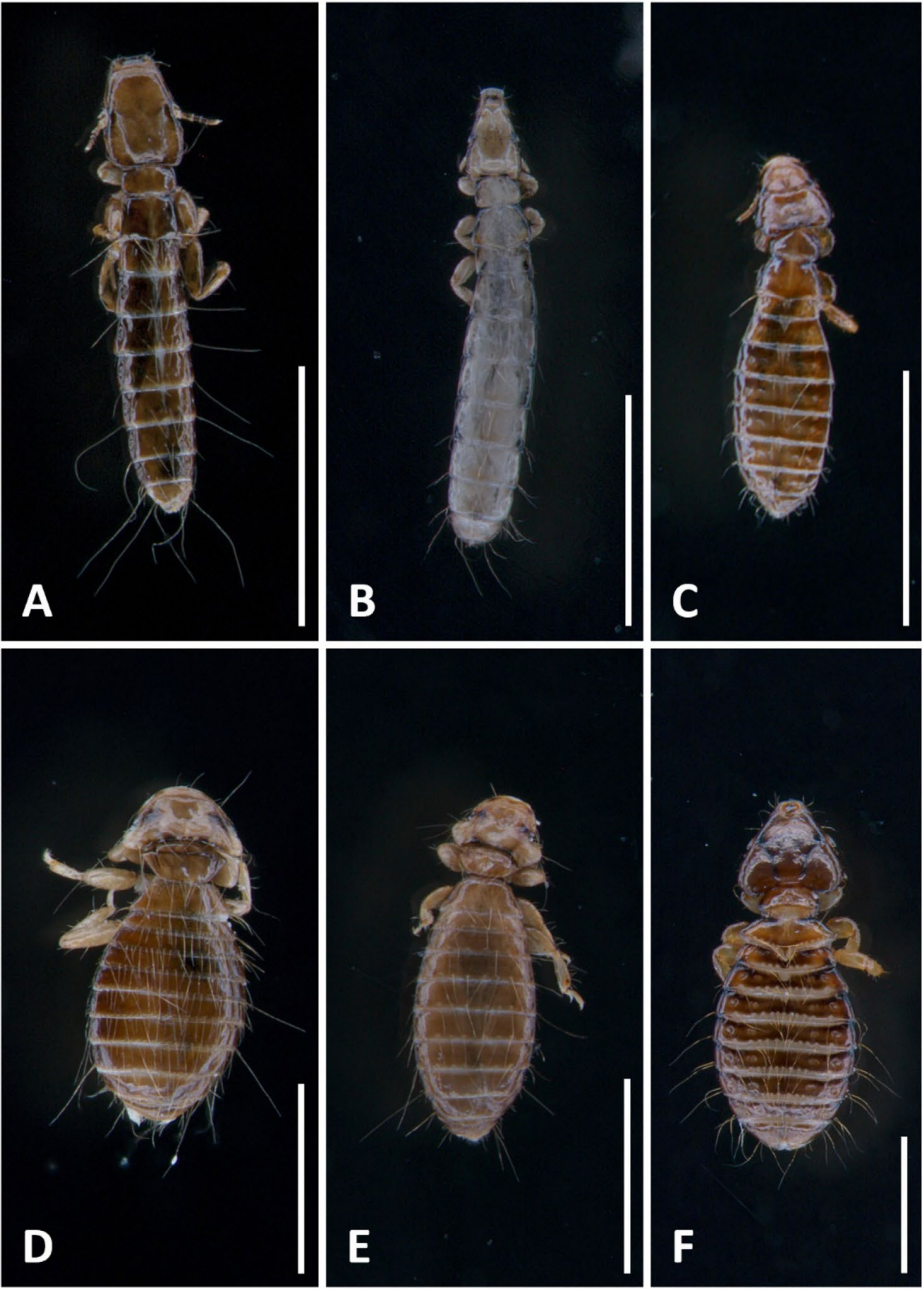
Examples of chewing lice collected from Charadriidae and Scolopacidae migrating through central Poland (one species per genus). A—*Rhynonirmus scolopacis*, B—*Quadraceps hiaticulae*, C—*Lunaceps schismatus*, D—*Austromenopon hystriculum*, E—*Actornithophilus ochraceus*, F—*Cummingsiella ovalis*. Scale bar = 1000 μm.

Due to the large scale of our screening of louse infestation in shorebirds, it was technically unfeasible to collect and identify all individuals (5002 louse specimens counted). For this reason, louse species identification was based on a subsample collected either from all infested host individuals (species with low sample sizes) or from ~25% infested host individuals (species with largest sample size, e.g., wood sandpiper and common snipe; Table 1). Using this subsampling scheme, we collected louse specimens from 236 shorebirds (approximately 16.6%) representing 17 species captured in 2019 and 2020. Between one and five adult louse specimens per bird were removed directly from wing feathers using thumb forceps and stored in Eppendorf tubes filled with 96% ethanol. Specimens were selected randomly for sampling, so that the proportions of louse species reported from this subsample reflected their true relative abundance. Identification employed a combination of techniques, including microscopic examination and DNA barcoding based on comparisons with the BOLD. Lice were identified under a light microscope using morphological and anatomical characters in a two‐step process conducted before and after DNA extraction. Identification followed local keys for Poland (Złotorzycka 1967, 1972, 1976, 1978, 1980, 1994), general family‐level keys (Price et al. 2003), and additional keys and species descriptions (Timmermann 1949a, 1949b, 1950, 1954, 1971; Hopkins and Timmermann 1954; Clay 1959, 1962; Gustafsson and Olsson 2012b). All lice selected for molecular identification were photographed for deposition in the BOLD database, and selected specimens from different genera were photographed for illustrative purposes in this study (Figure 1). Identified specimens have been deposited in the collection of the Department of Biodiversity Studies and Bioeducation, University of Lodz (Poland).

We selected 248 chewing louse specimens (ca. 5% of all counted lice) from 236 host birds for molecular identification, with up to three specimens representing each recognized morphotype per‐host. DNA was isolated from whole specimens using the Chelex protocol (Casquet et al. 2012). Amplification, quality control and sequencing of the standard gene region for animal DNA barcoding (COI‐cytochrome c oxidase subunit I; Hebert et al. 2003) was performed using Oxford Nanopore Technology (ONT), as described by Srivathsan et al. (2021, 2024). The COI region was amplified using the primer pair LCO1490‐JJ and HCO2198‐JJ (Astrin and Stüben 2008), each tagged with a 9 bp identifier (Srivathsan et al. 2024). Primer tags were designed using Barcode Generator (Comai and Howell 2012) in accordance with Srivathsan et al. (2019).

PCR was performed using the following thermal cycling protocol: an initial denaturation at 95°C for 5 min; 5 cycles of denaturation at 95°C for 50 s, annealing at 45°C for 50 s, and extension at 72°C for 60 s; 35 cycles of denaturation at 95°C for 50 s, annealing at 51°C for 50 s, and extension at 72°C for 60 s; and a final extension at 72°C for 2 min. A subset of PCR products was run on a 1% agarose gel to verify amplification success. Subsequently, 5 μL of each PCR product was pooled into a single 1.5 mL Eppendorf tube. The pooled sample was cleaned using Sera‐Mag Select (Cytiva LiveScience) and again checked on a 1% agarose gel to ensure purification success. Final DNA concentration was measured with a Qubit 4 Fluorometer using the Qubit dsDNA HS Assay Kit (Invitrogen by Thermo Fisher Scientific, Waltham, MA, USA).

The sequencing library was prepared using the Oxford Nanopore Ligation Sequencing Kit version V14 (SQK‐LSK114) according to the manufacturer's instructions and sequenced on a Flongle (R10.4.1; FLOFLG114) flow cell. Sequencing results were demultiplexed and DNA barcodes were generated using ONT Barcoder 2.0 (Srivathsan et al. 2024). All sequences were screened for potential contamination using BLAST (Altschul et al. 1990) and deposited in GenBank (https://www.ncbi.nlm.nih.gov/) under accession numbers PV175570–PV175753. Sequences were also submitted to BOLD to obtain Barcode Index Numbers (BINs), which cluster genetically similar sequences and serve as provisional species hypotheses (Ratnasingham and Hebert 2013). Voucher information, taxonomic classifications, photographs, and COI DNA barcode sequences are available in BOLD (dataset no. DS‐PLPHI; 10.5883/DS‐PLPHI) and these information are summarized in Table S1 in the Supporting Information.

Intraspecific and interspecific genetic distances were calculated using the Kimura 2‐parameter model (K2P; Kimura 1980) with the Barcode Gap Analysis tool available on the BOLD workbench. Because only a subsample of louse specimens from a relatively small proportion of host individuals was identified to species level, prevalence and abundance estimates for particular louse species could not be used in subsequent analyses. Consequently, all further analyses were conducted at the level of lice community, as our counts comprised different lice species, and the results should therefore be interpreted as reflecting the overall burden of chewing lice detected on the wings of shorebird hosts.

### 2.3 Statistical Analyses

We used generalized linear models (GLMs) to test for variation in relative louse prevalence and abundance (response variables) among host species, host age classes, sites, and years (all included as fixed factors). In addition, we used data from the two shorebird species with the largest sample sizes (wood sandpiper and common snipe) to examine associations between louse presence or abundance and host body condition. This analysis was performed separately for each of the two species. The measures of body condition (fat load and body mass) were entered as the response variable in separate models, whereas the presence or abundance of lice was included as predictor (fixed factor and linear covariate, respectively). To account for seasonal variation in louse occurrence and to control for variation in structural body size, date and wing length were included as two additional linear covariates in these models. Year was entered as a fixed factor. These analyses focused exclusively on first‐year (juvenile) individuals from the Jeziorsko site (*n* = 454 for wood sandpipers; *n* = 384 for common snipes), because sample sizes for both species were small at the Middle Vistula site and adult individuals were scarce at both locations. Individual (host) was treated as the unit of analysis in all the models, including models assessing interspecific variation in louse infestation.

All GLMs were performed using the *lme4* (Bates et al. 2015) and *car* (Fox and Weisberg 2011) packages in R. For louse presence, we used a binomial error distribution with a logit link function. For louse abundance, we used a Poisson error distribution with a log link function. Multicollinearity among predictors in all the models was low, as inferred from low variance inflation factors VIF < 2.00. Model assumptions were assessed using diagnostic plots generated with base R and the *performance* R package (Lüdecke et al. 2021). No issues with model convergence were detected. No model reduction (removal of nonsignificant predictors) was performed. Differences in louse presence and abundance among host species were evaluated using post hoc tests implemented in the *emmeans* package (Lenth et al. 2018). All values are presented as mean ± SE.

Between‐species variation in louse prevalence was visualized on the host phylogeny using the *phytools* package in R (Revell 2012). For the purpose of this visualization, phylogenetic relationships among shorebird hosts were reconstructed using the complete avian time‐calibrated phylogeny (Jetz et al. 2012) with the backbone tree developed by Ericson et al. (2006). To account for phylogenetic uncertainty, we downloaded 1000 alternative trees from the BirdTree webserver (www.birdtree.org; Jetz et al. 2012) and summarized them into a consensus phylogeny using Geneious version 10.0.5 (Biomatters Ltd., Auckland, New Zealand).

## 3 Results

### 3.1 Chewing Louse Species Composition and Diversity

In total, we identified 26 louse species collected from 236 host individuals representing 17 bird species (Table 1, Figure 1). The detected species belonged to two families: Ancistronidae (Amblycera; 9 species) and Philopteridae (Ischnocera; 17 species). Ancistronidae were represented by two genera: *Actornithophilus* (7 species) and *Austromenopon* (2 species), whereas Philopteridae included six genera: *Carduiceps* (1 species), *Cummingsiella* (1 species), *Lunaceps* (4 species), *Quadraceps* (9 species), *Rhynonirmus* (1 species), and *Saemundssonia* (1 species).

Per‐host louse diversity (species richness) ranged from one to five species across shorebird hosts. The mean diversity was 2.0 ± 0.9 species per individual. Of the 26 louse species recorded, 22 (85%) were found on a single host species (Table 1). Four species infested more than one host: *L. falcinellus* occurred on four different hosts, and *A. ochraceus* , *L. holophaeus*, and *Q. hiaticulae* were found on two host species each (Table 1).

We obtained 184 barcode sequences from chewing lice, corresponding to a 74% success rate from the initial 248 specimens selected for analysis (Table 3). These sequences originated from 23 species identified based on morphological characters; sequencing of specimens belonging to three additional species was unsuccessful for technical reasons. The mean number of sequences per species was 8.4 ± 14.6, with a maximum of 66 sequences for *Q. obscurus*, and seven species represented by a single sequence. The sequences were assigned to 23 BINs, five of which were unique, meaning that they formed distinct genetic clusters without overlap with any other BINs in the BOLD database. DNA barcodes within these unique BINs were sufficiently divergent from those in other clusters, which likely indicates distinct species or taxonomic units. All 23 louse species in our dataset were assigned to single BINs. Intraspecific genetic distances ranged from 0% to a maximum of 1.98% in *Actornithophilus totani* (BOLD: ACT8715), confirming the accurate assignment of barcode sequences to morphologically identified species (Table 3). Interestingly, the minimum K2P genetic distance to the nearest phylogenetic neighbor ranged from 12.69% to 31.98%, indicating substantial divergence among species. All COI barcodes represent the first sequences from the families Ancistronidae and Philopteridae originating from Poland. Furthermore, the sequences obtained for *Actornithophilus bicolor*, *A. flavipes* , *A. flumineus* , *A. pustulosus* , *Austromenopon durisetosum*, *Lunaceps falcinellus*, *L. schismatus*, *Quadraceps bicuspis*, *Rhynonirmus scolopacis*, and *Saemundssonia platygaster frater* constitute the first publicly available barcode references for these species.

**TABLE 3:** BOLD intraspecific genetic distance analysis for 23 chewing lice species collected from migrating shorebirds.

No.	Family	Species	N	BIN ID	Mean genetic distance (%)	Max. genetic distance (%)
1	Ancistronidae	*Actornithophilus bicolor*	1	AGE6447*	N/A	N/A
2	Ancistronidae	*Actornithophilus flavipes*	1	ACT8711	N/A	N/A
3	Ancistronidae	*Actornithophilus flumineus*	2	AGE6446*	0.92	0.92
4	Ancistronidae	*Actornithophilus ochraceus*	11	ADA8181	0.57	0.92
5	Ancistronidae	*Actornithophilus totani*	2	ACT8715	1.98	1.98
6	Ancistronidae	*Actornithophilus paludosus*	1	AGE6448*	N/A	N/A
7	Ancistronidae	*Actornithophilus pustulosus*	4	ACT8993	0.18	0.3
8	Ancistronidae	*Austromenopon durisetosum*	1	AGE5520*	N/A	N/A
9	Philopteridae	*Cummingsiella ovalis*	1	ADA6787	N/A	N/A
10	Philopteridae	*Lunaceps schismatus*	23	ACT8778	0.10	0.46
11	Philopteridae	*Lunaceps holophaeus*	5	ACT8860	0.12	0.31
12	Philopteridae	*Lunaceps numenii*	2	ACT8858	0.74	0.74
13	Philopteridae	*Lunaceps falcinellus*	7	ACT8776	0.13	0.31
14	Philopteridae	*Quadraceps bicuspis*	1	AGD1542*	N/A	N/A
15	Philopteridae	*Quadraceps furvus*	2	AFX5371	0.1	0.31
16	Philopteridae	*Quadraceps hiaticulae*	5	ACT8855	0	0
17	Philopteridae	*Quadraceps obscurus*	66	AFX5372	0.17	0.92
18	Philopteridae	*Quadraceps obtusus*	2	ACT8857	0.46	0.46
19	Philopteridae	*Quadraceps ravus*	18	ADA8030	0.13	0.46
20	Philopteridae	*Quadraceps similis*	5	AES5355	0.19	0.31
21	Philopteridae	*Quadraceps strepsilaris*	1	ADA7007	N/A	N/A
22	Philopteridae	*Rhynonirmus scolopacis*	22	AGD1392*	0.17	0.61
23	Philopteridae	*Saemundssonia platygaster*	1	AGC6064*	N/A	N/A

*Note:* N/A is displayed for intraspecific genetic distances in singleton species (only one sequence available); unique BINs are marked with asterisks.

Abbreviations: BIN, Barcode Index Number; *N*, number of sequences analyzed.

### 3.2 Variation Between Host Species and Age Classes

**FIGURE 2:**
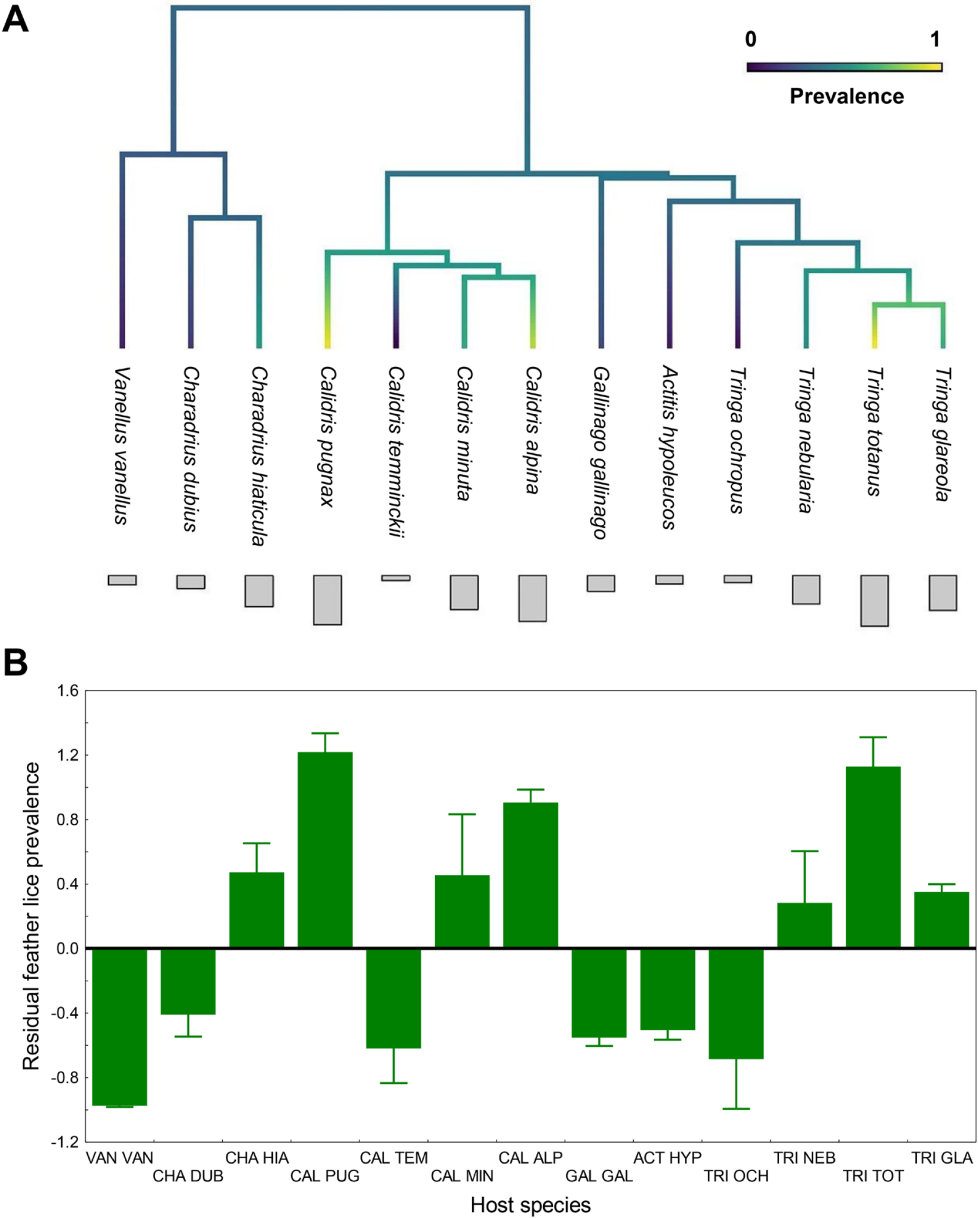
Variation in chewing louse prevalence among 13 shorebird species migrating through central Poland. Prevalence is shown as raw estimates plotted across the host (shorebird) phylogeny (A) and as mean (± SE) residual estimates from generalized linear models controlling for differences between age classes, sites, and years (B). Shorebird phylogenetic relationships were reconstructed using the complete time‐calibrated avian phylogeny developed by Jetz et al. (2012). Bars at the terminal branches of the phylogeny represent raw prevalence estimates (A).

**FIGURE 3:**
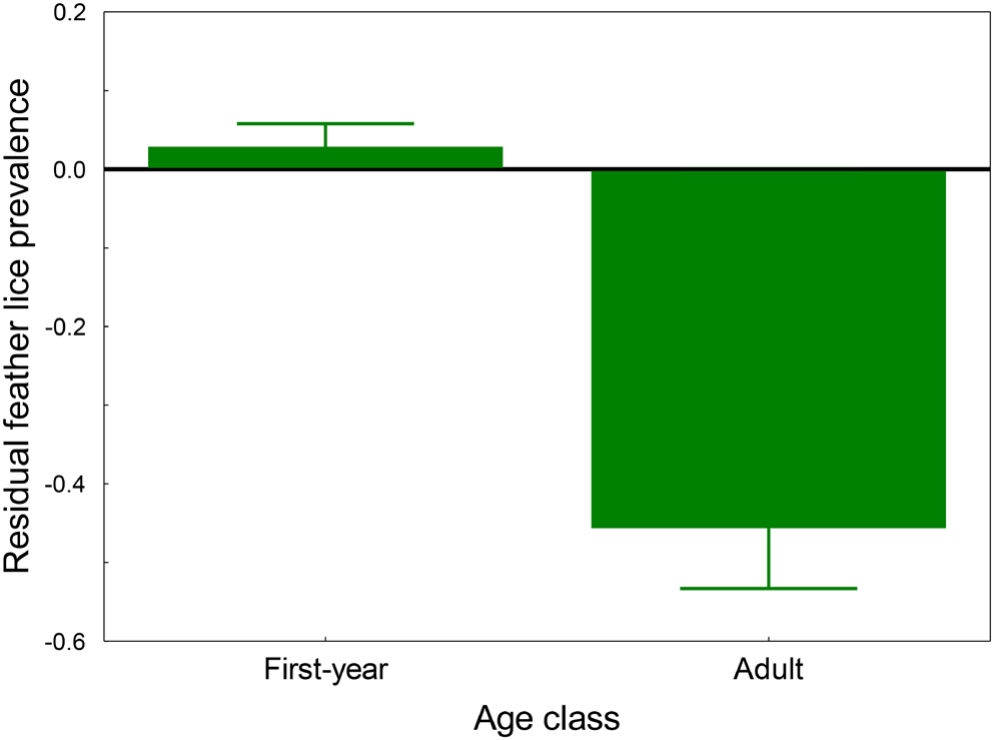
Variation in chewing louse prevalence between host age classes in shorebirds migrating through central Poland (all host species combined). Values represent mean (± SE) residual estimates from a generalized linear model controlling for variation among host species, sites, and years.

Infestation parameters varied considerably among host species. Prevalence ranged from 9% to 94%, and mean relative abundance ranged from 0.2 to 26.4 lice per individual, with mean infestation intensity ranging from 2.2 to 28.6 lice per infested bird (Table 2). Relatively low infestation (< 30% prevalence and < 1.0 abundance) was observed in six species ( *Actitis hypoleucos* , *Calidris temminckii* , *Charadrius dubius* , *Gallinago gallinago* , *Tringa ochropus* , and *Vanellus vanellus* ), whereas all remaining species showed relatively high prevalence (> 50%) and abundance (> 3.0) (Table 2; Figure 2). Generalized linear models confirmed that these differences in louse prevalence and abundance among host species were statistically significant (all *p* < 0.001; Table S2). Both prevalence and abundance differed significantly between host age classes, with adults exhibiting lower infestation levels than first‐year individuals (prevalence: β = −1.37 ± 0.29, *p* < 0.001; abundance: β = −1.04 ± 0.01, *p* < 0.001; Table S2; Figure 3). Significant differences between sites were also detected (prevalence: β = −1.11 ± 0.24, *p* < 0.001; abundance: β = −0.31 ± 0.06, *p* = 0.040; Table S2): shorebirds captured at the artificial reservoir had higher prevalence (0.49 ± 0.02) and abundance (3.9 ± 0.3) than those captured in the natural river valley (prevalence: 0.34 ± 0.03; abundance: 2.8 ± 0.4). In addition, louse prevalence and mean abundance varied significantly among years (all *p* < 0.001; Table S2).

### 3.3 Host Body Condition and Seasonal Variation

**FIGURE 4:**
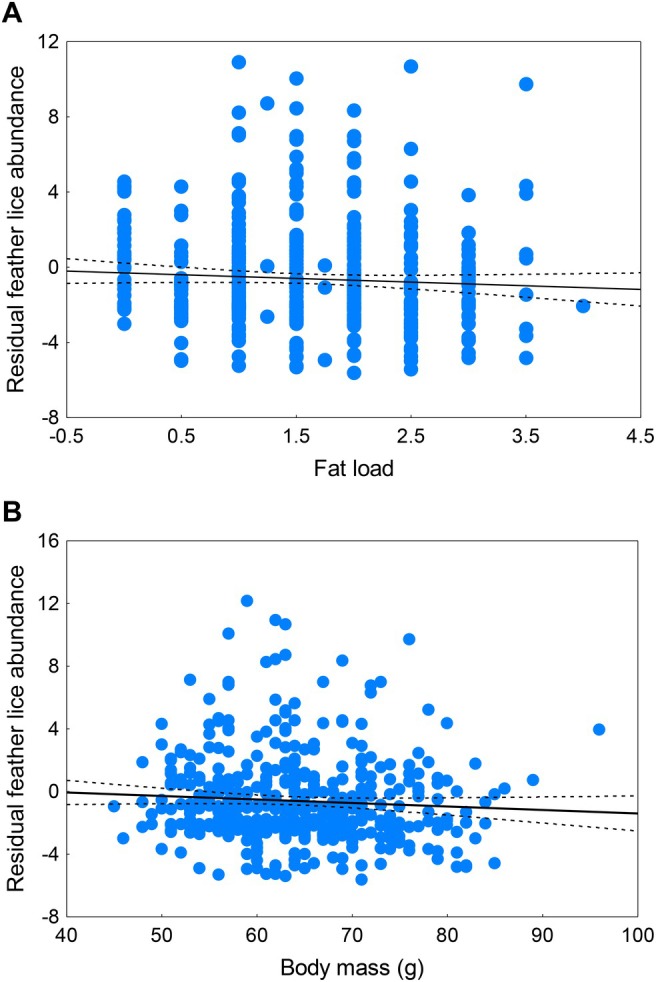
Associations between chewing louse abundance and body condition indices, i.e., fat load (A) and body mass (B), in first‐year wood sandpipers migrating through central Poland. Regression lines with 95% confidence intervals are shown for residual estimates from a generalized linear model.

The separate analyses of louse occurrence in two most common host species (wood sandpiper and common snipe) revealed that negative relationships of louse abundance with host body condition were apparent only in the wood sandpiper (fat load: β = −0.105 ± 0.024, *p* < 0.001; body mass: β = −0.012 ± 0.002, *p* < 0.001, Table S3, Figure 4). In contrast, no such relationships were detected in the common snipe (Table S3). Louse prevalence was not significantly related to host body condition in either species (Table S4). In the common snipe, both prevalence and abundance decreased significantly over the course of the migratory season (Tables S3 and S4). In contrast, louse abundance increased over the course of the migratory season in the wood sandpiper, whereas louse prevalence showed no significant seasonal pattern in this species (Tables S3 and S4).

## 4 Discussion

We demonstrated that chewing lice collected from the wings of shorebirds constitute a diverse ectoparasite community exhibiting considerable variation in host‐specific prevalence and abundance. Infestation rates differed with host age, being higher in young than in adult birds, and also varied between stopover sites, with higher values recorded at the artificial reservoir compared to the natural river valley. Furthermore, we found that chewing louse abundance was negatively associated with host body condition, although this relationship was evident only in the long‐distance migrant (wood sandpiper), but not in the short‐distance migrant (common snipe).

We provide new insights into the diversity and host associations of chewing lice parasitizing Charadriidae and Scolopacidae shorebirds. Our study updates the records of chewing lice in shorebirds migrating through Poland, including eight host species for which infestation levels were previously unknown (Złotorzycka et al. 1997). It also fills an important geographic gap, as previous data originated mainly from the Baltic coast and north‐western Poland, whereas systematic surveys had not been conducted along the major inland migration routes of the Warta and Vistula rivers (Złotorzycka et al. 1997; Złotorzycka and Modrzejewska 1988). In addition, we report two louse species for the first time in Poland (Złotorzycka et al. 1997; Złotorzycka and Modrzejewska 1988; Kadulski 2007): *A. flavipes* from the black‐bellied plover *Pluvialis squatarola* and *C. ovalis* from the Eurasian curlew *Numenius arquata* .

Molecular identification using DNA barcoding was successful and fully consistent with morphological species identification. At the same time, our study expands the barcode reference library for chewing lice by providing DNA barcode data for 23 species, including 10 species not previously represented in BOLD and 13 additional species with previous molecular records from outside Central Europe. As a result, our work increases the global DNA barcode reference library for chewing lice by approximately 5.6% and doubles the regional (Polish) DNA barcode reference library.

We found clear evidence that both louse prevalence and abundance were associated with host age. In our study, birds that had hatched only a few months before sampling (first‐year individuals) were more heavily infested than adult birds (older than 1 year). This pattern may be related to weaker antiparasite defenses in first‐year birds, including less effective preening and still‐developing immune responses, as well as to higher transmission opportunities during early life stages, when close contact with parents and other young birds may facilitate both vertical and horizontal transfer. Similar age‐related differences have been documented in pied flycatchers *Ficedula hypoleuca* , where louse prevalence and abundance decreased or fluctuated with age (Potti and Merino 1995), and in brown‐headed cowbirds *Molothrus ater* , where younger males exhibited higher louse prevalence than older males (Durkin et al. 2015). In cowbirds, this pattern was linked to behavioral differences, as younger individuals spent more time foraging in large groups, which likely increased opportunities for louse transmission (Durkin et al. 2015). However, several studies have reported no association between louse infestation levels and host age. For example, no differences were found between adults and nestlings in altricial species such as blackbirds *Turdus merula* (Brooke 2010) and European bee‐eaters *Merops apiaster* (Darolová et al. 2001). Likewise, similar infestation levels were observed in young (4–7 months old) and adult individuals of the precocial red‐legged partridge *Alectoris rufa* (Calvete et al. 2003). Such inconsistencies among studies may reflect differences in louse community composition and host life‐history traits (Galloway and Lamb 2021), including differences in the dominant developmental stages present on hosts (Broek 1967; Chandra et al. 1988) and in the timing of parasite transmission, which may occur soon after hatching in some host‐louse systems but only after the development of flight feathers in others (Broek 1967; Lee and Clayton 1995; Bartlett and Anderson 1989). Variation in local environmental conditions, particularly temperature and humidity (Agarwal and Saxena 1979; Lamb and Galloway 2019), as well as differences in sampling methods, may further contribute to differences in infestation estimates among host species and study sites.

We found that louse prevalence and abundance were also associated with the type of stopover habitat used by hosts during annual migration. Specifically, higher infestation levels were recorded in shorebirds captured at the artificial reservoir (Jeziorsko dam reservoir, Middle Warta River) compared to those from the natural river valley (Middle Vistula River). These habitat‐related differences in louse prevalence and abundance are most likely attributable to variation in the density and size of local host populations, which can influence opportunities for louse transmission and have been shown to correlate positively with infestation intensity (Clayton 1991; Tomás et al. 2016). The two study sites differed markedly in the availability of suitable foraging and roosting habitat. At the artificial reservoir, water level management during the autumn migration period creates highly favorable feeding conditions, resulting in large and dense aggregations of diverse shorebird flocks concentrated on relatively limited mudflat areas. In contrast, suitable foraging sites in the natural river valley are more widely dispersed, and shorebird flocks tend to be smaller and less diverse, which likely reduces the likelihood of louse transmission.

We observed contrasting seasonal dynamics of chewing louse occurrence in wood sandpipers and common snipes, two species that differ markedly in their migration strategies (Włodarczyk et al. 2007). It may be assumed that migratory behavior contributed to the differences observed between these two shorebird species. In wood sandpipers, louse abundance increased throughout the migratory period and was associated with lower fat load and body mass. In contrast, both prevalence and abundance decreased over the season in common snipes, and no relationship with body condition was detected. The wood sandpiper is a long‐distance migrant that travels to wintering areas in central Africa and follows a time‐minimizing migration strategy, which limits opportunities for refueling and preening during stopovers. Individuals in good condition typically depart early, whereas those in poorer condition (e.g., individuals originating from lower‐quality habitats or breeding sites with higher parasite pressure) may require longer stopovers to accumulate sufficient energy reserves and therefore depart later. As a result, birds migrating later in the season are likely to be in inferior condition and, as shown here, may also carry higher ectoparasite loads.

The common snipe follows a different migration strategy, characterized by relatively short distances between successive stopover sites and long stopover durations, which together contribute to a long migration period (Włodarczyk et al. 2007). Snipes overwinter relatively close to their breeding grounds, in Western Europe and the Mediterranean region (Minias et al. 2010), and their migration is therefore less time‐constrained compared to wood sandpipers. Consequently, common snipes are likely to maintain a relatively stable body condition throughout the migration period (energy‐minimization strategy), and strong seasonal declines in condition or ectoparasite load are not expected. In addition, many first‐year snipes begin post‐juvenile molt during migration and shed most wing feathers, including lesser and median coverts and tertials (Minias et al. 2010; Podlaszczuk et al. 2017). This extensive molt is likely to physically reduce the number of chewing lice as the season progresses and thereby diminish their potential impact on the host.

The abundance of lice collected from the wings was negatively correlated with fat load and body mass in the wood sandpiper, which likely reflects detrimental effects of relatively high parasite loads on host condition. Individuals carrying more lice appeared to incur greater energetic costs than those with lower infestation levels. An important question, however, is whether high infestation causes poor condition or whether individuals in poorer condition are more susceptible to higher infestation. Impaired condition may reduce the time available for preening and grooming, and reduced grooming effort can itself have a negative impact on condition (Ash 1960; Cotgreave and Clayton 1994). High louse loads have been shown to deteriorate host health, primarily by reducing thermal insulation and impairing flight efficiency (Booth et al. 1993; Barbosa et al. 2002). Increased parasite loads also require greater investment in preening, which may limit the time available for other activities, such as foraging, and thereby further reduce condition. Interestingly, we found no significant association between louse prevalence and host condition, suggesting that prevalence may be a less informative measure than abundance when evaluating potential fitness consequences of infestation. This may indicate that measurable biological effects of lice on hosts may become evident only when infestation levels are high.

Our correlative results suggest that chewing lice may negatively affect the condition of hosts with particular behavioral traits, such as migration strategy. Negative associations between chewing louse infestation and body condition were evident only in time‐constrained long‐distance migrants (wood sandpipers), but not in short‐distance migrants that experience fewer time limitations during migration (common snipes). The negative influence of ectoparasites, especially blood‐feeding taxa, on the condition and fitness of birds is well established (Clayton and Moore 1997; Pryor and Casto 2015; Nogueira et al. 2023). In contrast, the effects of feather‐feeding ectoparasites have received much less attention (Galloway and Lamb 2021). Although our study revealed negative associations between chewing louse abundance and host condition, these relationships occurred during a particularly demanding stage of the annual cycle. Migration requires high energetic investment and efficient flight performance, both of which may be compromised by chewing lice. It remains unknown whether such negative associations persist during other, potentially less demanding, periods of the annual cycle. It is also possible that the presence of these associations in only one host species, the long‐distance migrant, may be linked to differences in the composition of its feather‐louse community. Chewing lice collected from the wings of wood sandpipers and common snipes were dominated by species from two different genera (*Quadraceps obscurus* and *Rhynonirmus scolopacis*, respectively), which may exert contrasting effects on host fitness.

Many chewing louse species are highly specialized for particular regions of the host body. In our study, we were unlikely to detect or collect species that preferentially inhabit non‐wing areas, such as the head or torso, because these regions are covered by a dense layer of feathers that prevents effective visual inspection. As a result, lice associated with the wings, including both wing‐restricted taxa and taxa occupying a broader range of body regions, predominated in our dataset, and infestation parameters reflected only this specific ecological guild of lice. Although this represents a methodological limitation, restricting our efforts to a particular body region enabled a focused investigation of chewing lice that may have direct effects on the flight apparatus of their hosts.

Despite the advantages of our methodological approach, it is important to acknowledge its limitations. Visual inspection of host wings excludes non‐wing louse species from detection and even the counts of typical wing lice may be imperfect. It is likely that some proportion of wing lice were missed during inspection, particularly because they may temporarily move to other body regions (Harbison and Boughton 2014). Louse populations are typically aggregated, and most hosts carry only a few individuals, which increases the risk of overlooking them during visual examination. Movement of lice away from the wings may also be enhanced by host handling, such as ringing and collection of morphometric measurements (Vas and Fuisz 2010). Taking these factors into account, we recognize that our estimates of louse prevalence and abundance are likely to be underestimated. In addition, by restricting sampling to wings, our approach may also bias the representation of louse taxa and thus provide an incomplete picture of species composition on the host. Although fumigation procedures can provide more complete estimates of louse abundance (Clayton and Walther 2001), applying them on a broad scale within volunteer‐based bird ringing schemes would be difficult. Importantly, there is no a priori reason to expect that the underestimation associated with our visual protocol would differ systematically among species or among individuals within species. Together with the high repeatability of our counts, this suggests that the method is suitable for capturing relative inter‐ and intraspecific differences in infestation levels, even if absolute values should be interpreted with caution.

Another limitation of our study relates to the relatively narrow subsampling of louse specimens that were identified to species using a combination of microscopic and molecular approaches. Species identification was performed for approximately 5% of all recorded louse specimens, collected from around 16.6% of the inspected host individuals. Because our screening for louse prevalence and abundance was extensive, with more than five thousand lice counted across approximately 1400 hosts, identifying every specimen to species level would have required an immense investment of time and financial resources and was therefore not feasible. As a result, although our study provides a general characterization of louse species composition across diverse shorebird taxa and contributes new molecular data for these ectoparasites, our statistical analyses focused on the total burden of lice on the wings (all species combined). Therefore, in this study we interpret total louse abundance as a general measure of infestation intensity, while recognizing that different louse species may differ in their ecology and effects on hosts. We acknowledge that exploring fine‐scale, species‐specific effects of lice on avian fitness is an important and potentially insightful research direction. However, due to methodological constraints, such analyses fell outside the primary scope of the present study.

In conclusion, our study shows that the chewing lice living on the wings of shorebirds constitute a varied and strongly host‐associated ectoparasite group. Their prevalence and abundance vary widely among host species and are influenced by host age and the characteristics of stopover habitats. Importantly, we provided some of the first evidence that these feather‐feeding parasites are associated with host condition, and we suggest that this association may be linked to host migration strategy. We also contributed novel molecular barcode data that is likely to improve the reliability of species identification and enhance understanding of genetic diversity within this non‐model group of parasites. We recommend that future research should further investigate the composition of louse faunas across an even broader range of avian hosts to determine whether and how specific louse ecotypes and species influence the life histories of different bird taxa.

## Author Contributions

**Maciej Bartos:** conceptualization (lead), data curation (equal), formal analysis (equal), investigation (equal), methodology (equal), project administration (equal), resources (equal), supervision (lead), writing – original draft (lead), writing – review and editing (lead). **Radosław Włodarczyk:** conceptualization (equal), data curation (equal), formal analysis (equal), investigation (equal), methodology (equal), resources (equal), writing – original draft (supporting), writing – review and editing (equal). **Amelia Paszkowska:** investigation (supporting), writing – review and editing (equal). **Tomasz Rewicz:** data curation (equal), formal analysis (equal), investigation (equal), methodology (equal), project administration (supporting), resources (equal), supervision (equal), writing – original draft (supporting), writing – review and editing (equal). **Maciej Kamiński:** formal analysis (equal), investigation (equal), methodology (equal), writing – original draft (supporting), writing – review and editing (equal). **Jan Rapczyński:** investigation (equal), writing – review and editing (equal). **Patryk Fiutek:** investigation (equal), writing – review and editing (equal). **Piotr Minias:** conceptualization (lead), data curation (equal), formal analysis (equal), funding acquisition (lead), investigation (equal), methodology (equal), project administration (equal), resources (equal), supervision (lead), writing – original draft (supporting), writing – review and editing (lead).

## Funding

The Study Was Financially Supported by the National Science Center in Poland (Grant Number 2023/51/B/NZ8/01266).

## Ethics Statement

All procedures, including bird capture and ringing, were approved by the Regional and General Directorates for Environmental Protection.

## Conflicts of Interest

The authors declare no conflicts of interest.

## Supporting information


**Table S1:** Sample overview, host specimens, collection sites, sampling dates, and GenBank accession numbers.
**Table S2:** Models assessing variation in chewing louse infestation traits (prevalence and abundance) among host species, host age classes, sites, and years in shorebirds migrating through central Poland. Significant effects are indicated in bold.
**Table S3:** Models assessing relationships between chewing louse abundance and host body condition (fat load and body mass) in first‐year wood sandpipers and common snipes migrating through central Poland. Significant effects are indicated in bold.
**Table S4:** Models assessing relationships between chewing louse prevalence and host body condition (fat load and body mass) in first‐year wood sandpipers and common snipes migrating through central Poland. Significant effects are indicated in bold.

**Data S1:** The molecular dataset generated during this study, together with the associated metadata, is available in the DS‐PLPHI dataset (10.5883/DS‐PLPHI) in the public repository of the Barcode of Life Data Systems and in GenBank (accession numbers PV175570–PV175753; https://www.ncbi.nlm.nih.gov/genbank). Raw data: Bartos et al. Raw Data.

## Data Availability

The data that supports the findings of this study are available in the Supporting Information of this article.
