# Supplementary material for: Chewing Lice From the Wings of Migrating Shorebirds: Diversity, Host Specificity, and Associations With Host Body Condition
Source: Ecol Evol. 2026 Jul 25;16(7):e74013. doi: 10.1002/ece3.74013 (PMC13401268; doi:10.1002/ece3.74013)
Supplement: Supplementary file 1 — Table S1: Sample overview, host specimens, collection sites, sampling dates, and GenBank accession numbers. Table S2: Models assessing variation in chewing louse infestation traits (prevalence and abundance) among host species, host age classes, sites, and years in shorebirds migrating through central Poland. Significant effects are indicated in bold. Table S3: Models assessing relationships between chewing louse abundance and host body condition (fat load and body mass) in first‐year wood sandpipers and common snipes migrating through central Poland. Significant effects are indicated in bold. Table S4: Models assessing relationships between chewing louse prevalence and host body condition (fat load and body mass) in first‐year wood sandpipers and common snipes migrating through central Poland. Significant effects are indicated in bold. [file ECE3-16-e74013-s002.docx]

**Supporting Information**

**Table S1.** Sample overview, host specimens, collection sites, sampling dates, and GenBank accession numbers.

| **Identification** | **Process ID** | **BIN** | **GenBank accession number** | **Catalog number** | **Associated taxa** | **Associated specimens** |
| --- | --- | --- | --- | --- | --- | --- |
| Rhynonirmus scolopacis | PLPHI001-24 | N/A | N/A | PHILPL_0001 | Host: Gallinago gallinago | HN61992 |
| Rhynonirmus scolopacis | PLPHI002-24 | N/A | N/A | PHILPL_0002 | Host: Gallinago gallinago | HN62002 |
| Rhynonirmus scolopacis | PLPHI004-24 | BOLD:AGD1392 | PV175659 | PHILPL_0004 | Host: Gallinago gallinago | HN62041 |
| Rhynonirmus scolopacis | PLPHI006-24 | BOLD:AGD1392 | PV175626 | PHILPL_0006 | Host: Gallinago gallinago | HN62010 |
| Rhynonirmus scolopacis | PLPHI008-24 | N/A | N/A | PHILPL_0008 | Host: Gallinago gallinago | HN62000 |
| Rhynonirmus scolopacis | PLPHI009-24 | N/A | N/A | PHILPL_0009 | Host: Gallinago gallinago | HN62001 |
| Rhynonirmus scolopacis | PLPHI010-24 | N/A | N/A | PHILPL_0010 | Host: Gallinago gallinago | HN61993 |
| Rhynonirmus scolopacis | PLPHI013-24 | BOLD:AGD1392 | PV175579 | PHILPL_0013 | Host: Gallinago gallinago | HN62015 |
| Rhynonirmus scolopacis | PLPHI014-24 | BOLD:AGD1392 | PV175585 | PHILPL_0014 | Host: Gallinago gallinago | HN61977 |
| Rhynonirmus scolopacis | PLPHI015-24 | BOLD:AGD1392 | PV175725 | PHILPL_0015 | Host: Gallinago gallinago | HN61982 |
| Rhynonirmus scolopacis | PLPHI016-24 | BOLD:AGD1392 | PV175741 | PHILPL_0016 | Host: Gallinago gallinago | HN61992 |
| Rhynonirmus scolopacis | PLPHI017-24 | BOLD:AGD1392 | PV175593 | PHILPL_0017 | Host: Gallinago gallinago | HN61966 |
| Rhynonirmus scolopacis | PLPHI018-24 | N/A | N/A | PHILPL_0018 | Host: Gallinago gallinago | HN61788 |
| Rhynonirmus scolopacis | PLPHI019-24 | BOLD:AGD1392 | PV175748 | PHILPL_0019 | Host: Gallinago gallinago | HN61961 |
| Rhynonirmus scolopacis | PLPHI022-24 | BOLD:AGD1392 | PV175622 | PHILPL_0022 | Host: Gallinago gallinago | HN61763 |
| Rhynonirmus scolopacis | PLPHI023-24 | BOLD:AGD1392 | PV175573 | PHILPL_0023 | Host: Gallinago gallinago | HN61772 |
| Rhynonirmus scolopacis | PLPHI024-24 | BOLD:AGD1392 | PV175589 | PHILPL_0024 | Host: Gallinago gallinago | HN61955 |
| Rhynonirmus scolopacis | PLPHI025-24 | BOLD:AGD1392 | PV175703 | PHILPL_0025 | Host: Gallinago gallinago | HN61950 |
| Rhynonirmus scolopacis | PLPHI027-24 | BOLD:AGD1392 | PV175696 | PHILPL_0027 | Host: Gallinago gallinago | HN61956 |
| Rhynonirmus scolopacis | PLPHI028-24 | N/A | N/A | PHILPL_0028 | Host: Gallinago gallinago | HN62016 |
| Rhynonirmus scolopacis | PLPHI029-24 | BOLD:AGD1392 | PV175680 | PHILPL_0029 | Host: Gallinago gallinago | HN61960 |
| Austromenopon durisetosum | PLPHI032-24 | BOLD:AGE5520 | PV175594 | PHILPL_0032 | Host: Gallinago gallinago | HN62119 |
| Quadraceps ravus | PLPHI033-24 | N/A | N/A | PHILPL_0033 | Host: Actitis hypoleucos | JT35607 |
| Quadraceps ravus | PLPHI036-24 | BOLD:ADA8030 | PV175707 | PHILPL_0036 | Host: Actitis hypoleucos | JT35603 |
| Quadraceps ravus | PLPHI037-24 | BOLD:ADA8030 | PV175682 | PHILPL_0037 | Host: Actitis hypoleucos | JT35619 |
| Quadraceps hiaticulae | PLPHI038-24 | BOLD:ACT8855 | PV175719 | PHILPL_0038 | Host: Actitis hypoleucos | JT25391 |
| Actornithophilus flumineus | PLPHI039-24 | N/A | N/A | PHILPL_0039 | Host: Actitis hypoleucos | JT25391 |
| Quadraceps ravus | PLPHI040-24 | N/A | N/A | PHILPL_0040 | Host: Actitis hypoleucos | JT25380 |
| Quadraceps ravus | PLPHI041-24 | N/A | N/A | PHILPL_0041 | Host: Actitis hypoleucos | JT25386 |
| Quadraceps ravus | PLPHI043-24 | N/A | N/A | PHILPL_0043 | Host: Actitis hypoleucos | YN17451 |
| Quadraceps ravus | PLPHI044-24 | N/A | N/A | PHILPL_0044 | Host: Actitis hypoleucos | JT25374 |
| Quadraceps obscurus | PLPHI047-24 | BOLD:AFX5372 | PV175587 | PHILPL_0047 | Host: Tringa glareola | TS33500 |
| Quadraceps obscurus | PLPHI049-24 | BOLD:AFX5372 | PV175648 | PHILPL_0049 | Host: Tringa glareola | TS33497 |
| Quadraceps obscurus | PLPHI054-24 | BOLD:AFX5372 | PV175684 | PHILPL_0054 | Host: Tringa glareola | TS33488 |
| Quadraceps obscurus | PLPHI055-24 | BOLD:AFX5372 | PV175651 | PHILPL_0055 | Host: Tringa glareola | TS33382 |
| Quadraceps obscurus | PLPHI058-24 | BOLD:AFX5372 | PV175581 | PHILPL_0058 | Host: Tringa glareola | TS33481 |
| Quadraceps obscurus | PLPHI061-24 | BOLD:AFX5372 | PV175620 | PHILPL_0061 | Host: Tringa glareola | TS33485 |
| Quadraceps obscurus | PLPHI064-24 | BOLD:AFX5372 | PV175677 | PHILPL_0064 | Host: Tringa glareola | TS33491 |
| Quadraceps obscurus | PLPHI067-24 | BOLD:AFX5372 | PV175610 | PHILPL_0067 | Host: Tringa glareola | TS33490 |
| Quadraceps obscurus | PLPHI070-24 | BOLD:AFX5372 | PV175704 | PHILPL_0070 | Host: Tringa glareola | TS33489 |
| Quadraceps obscurus | PLPHI071-24 | BOLD:AFX5372 | PV175600 | PHILPL_0071 | Host: Tringa glareola | TS33507 |
| Quadraceps obscurus | PLPHI074-24 | BOLD:AFX5372 | PV175608 | PHILPL_0074 | Host: Tringa glareola | TS33483 |
| Quadraceps obscurus | PLPHI076-24 | BOLD:AFX5372 | PV175578 | PHILPL_0076 | Host: Tringa glareola | TS33213 |
| Quadraceps obscurus | PLPHI079-24 | BOLD:AFX5372 | PV175654 | PHILPL_0079 | Host: Tringa glareola | TS33486 |
| Quadraceps obscurus | PLPHI082-24 | BOLD:AFX5372 | PV175730 | PHILPL_0082 | Host: Tringa glareola | TS33501 |
| Quadraceps obscurus | PLPHI084-24 | BOLD:AFX5372 | PV175628 | PHILPL_0084 | Host: Tringa glareola | TS33475 |
| Quadraceps obscurus | PLPHI087-24 | BOLD:AFX5372 | PV175670 | PHILPL_0087 | Host: Tringa glareola | TS33484 |
| Quadraceps obscurus | PLPHI090-24 | BOLD:AFX5372 | PV175727 | PHILPL_0090 | Host: Tringa glareola | TS33503 |
| Quadraceps obscurus | PLPHI093-24 | BOLD:AFX5372 | PV175706 | PHILPL_0093 | Host: Tringa glareola | TS33426 |
| Quadraceps obscurus | PLPHI094-24 | BOLD:AFX5372 | PV175601 | PHILPL_0094 | Host: Tringa glareola | TS33451 |
| Quadraceps obscurus | PLPHI095-24 | BOLD:AFX5372 | PV175665 | PHILPL_0095 | Host: Tringa glareola | TS33452 |
| Quadraceps obscurus | PLPHI098-24 | BOLD:AFX5372 | PV175715 | PHILPL_0098 | Host: Tringa glareola | TS33455 |
| Quadraceps obscurus | PLPHI100-24 | BOLD:AFX5372 | PV175753 | PHILPL_0100 | Host: Tringa glareola | TS33453 |
| Quadraceps obscurus | PLPHI103-24 | BOLD:AFX5372 | PV175673 | PHILPL_0103 | Host: Tringa glareola | TS33443 |
| Quadraceps obscurus | PLPHI106-24 | N/A | N/A | PHILPL_0106 | Host: Tringa glareola | TS33456 |
| Quadraceps obscurus | PLPHI109-24 | BOLD:AFX5372 | PV175639 | PHILPL_0109 | Host: Tringa glareola | TS33478 |
| Quadraceps obscurus | PLPHI112-24 | N/A | N/A | PHILPL_0112 | Host: Tringa glareola | TS33480 |
| Quadraceps obscurus | PLPHI113-24 | BOLD:AFX5372 | PV175667 | PHILPL_0113 | Host: Tringa glareola | TS33457 |
| Quadraceps obscurus | PLPHI116-24 | BOLD:AFX5372 | PV175652 | PHILPL_0116 | Host: Tringa glareola | TS33621 |
| Quadraceps obscurus | PLPHI119-24 | BOLD:AFX5372 | PV175669 | PHILPL_0119 | Host: Tringa glareola | TS33487 |
| Quadraceps obscurus | PLPHI122-24 | BOLD:AFX5372 | PV175591 | PHILPL_0122 | Host: Tringa glareola | TS33454 |
| Quadraceps obscurus | PLPHI125-24 | BOLD:AFX5372 | PV175739 | PHILPL_0125 | Host: Tringa glareola | TS33450 |
| Quadraceps obscurus | PLPHI126-24 | BOLD:AFX5372 | PV175655 | PHILPL_0126 | Host: Tringa glareola | TS33516 |
| Quadraceps obscurus | PLPHI127-24 | BOLD:AFX5372 | PV175713 | PHILPL_0127 | Host: Tringa glareola | TS33496 |
| Quadraceps obscurus | PLPHI128-24 | BOLD:AFX5372 | PV175597 | PHILPL_0128 | Host: Tringa glareola | TS33515 |
| Quadraceps obscurus | PLPHI130-24 | BOLD:AFX5372 | PV175635 | PHILPL_0130 | Host: Tringa glareola | TS33531 |
| Quadraceps obscurus | PLPHI131-24 | BOLD:AFX5372 | PV175605 | PHILPL_0131 | Host: Tringa glareola | TS33528 |
| Quadraceps obscurus | PLPHI134-24 | BOLD:AFX5372 | PV175588 | PHILPL_0134 | Host: Tringa glareola | TS53512 |
| Quadraceps obscurus | PLPHI137-24 | BOLD:AFX5372 | PV175638 | PHILPL_0137 | Host: Tringa glareola | TS33477 |
| Quadraceps obscurus | PLPHI140-24 | BOLD:AFX5372 | PV175672 | PHILPL_0140 | Host: Tringa glareola | TS33505 |
| Quadraceps obscurus | PLPHI143-24 | BOLD:AFX5372 | PV175691 | PHILPL_0143 | Host: Tringa glareola | TS33523 |
| Quadraceps obscurus | PLPHI146-24 | BOLD:AFX5372 | PV175683 | PHILPL_0146 | Host: Tringa glareola | TS33533 |
| Quadraceps obscurus | PLPHI149-24 | BOLD:AFX5372 | PV175685 | PHILPL_0149 | Host: Tringa glareola | N/A |
| Quadraceps obscurus | PLPHI152-24 | BOLD:AFX5372 | PV175674 | PHILPL_0152 | Host: Tringa glareola | TS33535 |
| Quadraceps obscurus | PLPHI155-24 | BOLD:AFX5372 | PV175633 | PHILPL_0155 | Host: Tringa glareola | TS33520 |
| Quadraceps obscurus | PLPHI158-24 | BOLD:AFX5372 | PV175598 | PHILPL_0158 | Host: Tringa glareola | TS33530 |
| Quadraceps obscurus | PLPHI159-24 | N/A | N/A | PHILPL_0159 | Host: Tringa glareola | TS53511 |
| Quadraceps obscurus | PLPHI162-24 | BOLD:AFX5372 | PV175692 | PHILPL_0162 | Host: Tringa glareola | TS33534 |
| Quadraceps obscurus | PLPHI165-24 | BOLD:AFX5372 | PV175615 | PHILPL_0165 | Host: Tringa glareola | TS33372 |
| Quadraceps obscurus | PLPHI167-24 | BOLD:AFX5372 | PV175738 | PHILPL_0167 | Host: Tringa glareola | TS33506 |
| Quadraceps obscurus | PLPHI170-24 | BOLD:AFX5372 | PV175618 | PHILPL_0170 | Host: Tringa glareola | TS53510 |
| Quadraceps obscurus | PLPHI171-24 | BOLD:AFX5372 | PV175571 | PHILPL_0171 | Host: Tringa glareola | TS33494 |
| Quadraceps obscurus | PLPHI173-24 | BOLD:AFX5372 | PV175699 | PHILPL_0173 | Host: Tringa glareola | TS33493 |
| Lunaceps holophaeus | PLPHI176-24 | N/A | N/A | PHILPL_0176 | Host: Calidris pugnax | GN21481 |
| Lunaceps holophaeus | PLPHI179-24 | N/A | N/A | PHILPL_0179 | Host: Calidris pugnax | GN21485 |
| Actornithophilus pustulosus | PLPHI182-24 | BOLD:ACT8993 | PV175663 | PHILPL_0182 | Host: Calidris pugnax | HN61996 |
| Actornithophilus pustulosus | PLPHI184-24 | N/A | N/A | PHILPL_0184 | Host: Calidris pugnax | HN65520 |
| Lunaceps holophaeus | PLPHI186-24 | N/A | N/A | PHILPL_0186 | Host: Calidris pugnax | GN21487 |
| Actornithophilus pustulosus | PLPHI188-24 | BOLD:ACT8993 | PV175623 | PHILPL_0188 | Host: Calidris pugnax | GN21487 |
| Quadraceps obtusus | PLPHI189-24 | BOLD:ACT8857 | PV175679 | PHILPL_0189 | Host: Tringa totanus | GN21480 |
| Quadraceps obtusus | PLPHI191-24 | N/A | N/A | PHILPL_0191 | Host: Tringa totanus | GN21489 |
| Actornithophilus totani | PLPHI193-24 | BOLD:ACT8715 | PV175647 | PHILPL_0193 | Host: Tringa totanus | GN21489 |
| Quadraceps obtusus | PLPHI194-24 | N/A | N/A | PHILPL_0194 | Host: Tringa totanus | GN21486 |
| Actornithophilus totani | PLPHI197-24 | BOLD:ACT8715 | PV175645 | PHILPL_0197 | Host: Tringa totanus | GN21486 |
| Quadraceps ochropi | PLPHI200-24 | N/A | N/A | PHILPL_0200 | Host: Tringa ochropus | HN61968 |
| Actornithophilus paludosus | PLPHI205-24 | BOLD:AGE6448 | PV175709 | PHILPL_0205 | Host: Tringa nebularia | N/A |
| Quadraceps strepsilaris | PLPHI206-24 | BOLD:ADA7007 | PV175687 | PHILPL_0206 | Host: Arenaria interpres | HN61976 |
| Actornithophilus bicolor | PLPHI210-24 | BOLD:AGE6447 | PV175619 | PHILPL_0210 | Host: Arenaria interpres | HN61976 |
| Lunaceps numenii | PLPHI214-24 | BOLD:ACT8858 | PV175632 | PHILPL_0214 | Host: Numenius arquata | EA02959 |
| Rhynonirmus scolopacis | PLPHI217-24 | BOLD:AGD1392 | PV175606 | PHILPL_0217 | Host: Gallinago gallinago | HN24687 |
| Rhynonirmus scolopacis | PLPHI218-24 | N/A | N/A | PHILPL_0218 | Host: Gallinago gallinago | N/A |
| Rhynonirmus scolopacis | PLPHI219-24 | BOLD:AGD1392 | PV175678 | PHILPL_0219 | Host: Gallinago gallinago | HN24695 |
| Rhynonirmus scolopacis | PLPHI220-24 | BOLD:AGD1392 | PV175731 | PHILPL_0220 | Host: Gallinago gallinago | HN24696 |
| Rhynonirmus scolopacis | PLPHI221-24 | BOLD:AGD1392 | PV175646 | PHILPL_0221 | Host: Gallinago gallinago | HN24672 |
| Rhynonirmus scolopacis | PLPHI222-24 | BOLD:AGD1392 | PV175596 | PHILPL_0222 | Host: Gallinago gallinago | HN65511 |
| Rhynonirmus scolopacis | PLPHI225-24 | N/A | N/A | PHILPL_0225 | Host: Gallinago gallinago | HN65514 |
| Rhynonirmus scolopacis | PLPHI226-24 | BOLD:AGD1392 | PV175607 | PHILPL_0226 | Host: Gallinago gallinago | HN24689 |
| Rhynonirmus scolopacis | PLPHI227-24 | BOLD:AGD1392 | PV175627 | PHILPL_0227 | Host: Gallinago gallinago | HN65501 |
| Rhynonirmus scolopacis | PLPHI229-24 | BOLD:AGD1392 | PV175744 | PHILPL_0229 | Host: Gallinago gallinago | HN24691 |
| Rhynonirmus scolopacis | PLPHI230-24 | N/A | N/A | PHILPL_0230 | Host: Gallinago gallinago | HN65527 |
| Rhynonirmus scolopacis | PLPHI231-24 | N/A | N/A | PHILPL_0231 | Host: Gallinago gallinago | HN65541 |
| Quadraceps ravus | PLPHI232-24 | BOLD:ADA8030 | PV175749 | PHILPL_0232 | Host: Actitis hypoleucos | JN17450 |
| Quadraceps ravus | PLPHI235-24 | BOLD:ADA8030 | PV175690 | PHILPL_0235 | Host: Actitis hypoleucos | JT80621 |
| Quadraceps ravus | PLPHI236-24 | N/A | N/A | PHILPL_0236 | Host: Actitis hypoleucos | JT79559 |
| Quadraceps ravus | PLPHI237-24 | BOLD:ADA8030 | PV175583 | PHILPL_0237 | Host: Actitis hypoleucos | JT79683 |
| Quadraceps ravus | PLPHI238-24 | BOLD:ADA8030 | PV175701 | PHILPL_0238 | Host: Actitis hypoleucos | JT79606 |
| Quadraceps ravus | PLPHI239-24 | BOLD:ADA8030 | PV175631 | PHILPL_0239 | Host: Actitis hypoleucos | JT79586 |
| Quadraceps ravus | PLPHI242-24 | BOLD:ADA8030 | PV175592 | PHILPL_0242 | Host: Actitis hypoleucos | JT79527 |
| Actornithophilus flumineus | PLPHI245-24 | BOLD:AGE6446 | PV175661 | PHILPL_0245 | Host: Actitis hypoleucos | JT79685 |
| Quadraceps ravus | PLPHI248-24 | BOLD:ADA8030 | PV175694 | PHILPL_0248 | Host: Actitis hypoleucos | JT79689 |
| Quadraceps ravus | PLPHI249-24 | BOLD:ADA8030 | PV175624 | PHILPL_0249 | Host: Actitis hypoleucos | JT79591 |
| Quadraceps ravus | PLPHI250-24 | BOLD:ADA8030 | PV175752 | PHILPL_0250 | Host: Actitis hypoleucos | JT79671 |
| Quadraceps ravus | PLPHI251-24 | BOLD:ADA8030 | PV175751 | PHILPL_0251 | Host: Actitis hypoleucos | JT79690 |
| Quadraceps ravus | PLPHI252-24 | BOLD:ADA8030 | PV175660 | PHILPL_0252 | Host: Actitis hypoleucos | JT79673 |
| Quadraceps ravus | PLPHI253-24 | BOLD:ADA8030 | PV175728 | PHILPL_0253 | Host: Actitis hypoleucos | JT79674 |
| Actornithophilus flumineus | PLPHI254-24 | BOLD:AGE6446 | PV175734 | PHILPL_0254 | Host: Actitis hypoleucos | JT79678 |
| Quadraceps ravus | PLPHI255-24 | BOLD:ADA8030 | PV175641 | PHILPL_0255 | Host: Actitis hypoleucos | JT79636 |
| Quadraceps ravus | PLPHI256-24 | BOLD:ADA8030 | PV175714 | PHILPL_0256 | Host: Actitis hypoleucos | JT79325 |
| Quadraceps ravus | PLPHI257-24 | BOLD:ADA8030 | PV175695 | PHILPL_0257 | Host: Actitis hypoleucos | JT79688 |
| Austromenopon hystriculum | PLPHI260-24 | N/A | N/A | PHILPL_0260 | Host: Actitis hypoleucos | JT79541 |
| Actornithophilus flumineus | PLPHI261-24 | N/A | N/A | PHILPL_0261 | Host: Actitis hypoleucos | JT79687 |
| Quadraceps ravus | PLPHI262-24 | BOLD:ADA8030 | PV175708 | PHILPL_0262 | Host: Actitis hypoleucos | JT79669 |
| Quadraceps obscurus | PLPHI263-24 | BOLD:AFX5372 | PV175621 | PHILPL_0263 | Host: Tringa glareola | TS44167 |
| Quadraceps obscurus | PLPHI266-24 | BOLD:AFX5372 | PV175721 | PHILPL_0266 | Host: Tringa glareola | TS44158 |
| Quadraceps obscurus | PLPHI269-24 | BOLD:AFX5372 | PV175644 | PHILPL_0269 | Host: Tringa glareola | TS44170 |
| Quadraceps obscurus | PLPHI272-24 | N/A | N/A | PHILPL_0272 | Host: Tringa glareola | TS44168 |
| Quadraceps obscurus | PLPHI275-24 | BOLD:AFX5372 | PV175570 | PHILPL_0275 | Host: Tringa glareola | TS44131 |
| Quadraceps obscurus | PLPHI276-24 | BOLD:AFX5372 | PV175671 | PHILPL_0276 | Host: Tringa glareola | TS44122 |
| Quadraceps obscurus | PLPHI277-24 | BOLD:AFX5372 | PV175740 | PHILPL_0277 | Host: Tringa glareola | TS44159 |
| Quadraceps obscurus | PLPHI280-24 | BOLD:AFX5372 | PV175664 | PHILPL_0280 | Host: Tringa glareola | TS44165 |
| Quadraceps obscurus | PLPHI283-24 | BOLD:AFX5372 | PV175595 | PHILPL_0283 | Host: Tringa glareola | TS44172 |
| Quadraceps obscurus | PLPHI284-24 | BOLD:AFX5372 | PV175574 | PHILPL_0284 | Host: Tringa glareola | TS44164 |
| Quadraceps obscurus | PLPHI285-24 | BOLD:AFX5372 | PV175599 | PHILPL_0285 | Host: Tringa glareola | TS44161 |
| Quadraceps obscurus | PLPHI286-24 | BOLD:AFX5372 | PV175742 | PHILPL_0286 | Host: Tringa glareola | TS35202 |
| Lunaceps falcinellus | PLPHI287-24 | BOLD:ACT8776 | PV175700 | PHILPL_0287 | Host: Tringa glareola | TS35225 |
| Quadraceps obscurus | PLPHI288-24 | N/A | N/A | PHILPL_0288 | Host: Tringa glareola | N/A |
| Quadraceps obscurus | PLPHI289-24 | BOLD:AFX5372 | PV175636 | PHILPL_0289 | Host: Tringa glareola | TS35215 |
| Quadraceps obscurus | PLPHI292-24 | BOLD:AFX5372 | PV175666 | PHILPL_0292 | Host: Tringa glareola | TS35211 |
| Quadraceps obscurus | PLPHI294-24 | N/A | PV175711 | PHILPL_0294 | Host: Tringa glareola | TS35201 |
| Quadraceps obscurus | PLPHI295-24 | N/A | N/A | PHILPL_0295 | Host: Tringa glareola | TS35208 |
| Quadraceps obscurus | PLPHI298-24 | BOLD:AFX5372 | PV175611 | PHILPL_0298 | Host: Tringa glareola | TS35210 |
| Quadraceps obscurus | PLPHI301-24 | BOLD:AFX5372 | PV175743 | PHILPL_0301 | Host: Tringa glareola | TS35209 |
| Quadraceps obscurus | PLPHI302-24 | BOLD:AFX5372 | PV175712 | PHILPL_0302 | Host: Tringa glareola | TS35241 |
| Lunaceps holophaeus | PLPHI305-24 | BOLD:ACT8860 | PV175720 | PHILPL_0305 | Host: Calidris pugnax | HN24698 |
| Actornithophilus pustulosus | PLPHI308-24 | BOLD:ACT8993 | PV175577 | PHILPL_0308 | Host: Calidris pugnax | HN65534 |
| Lunaceps holophaeus | PLPHI309-24 | N/A | N/A | PHILPL_0309 | Host: Calidris pugnax | GN27884 |
| Actornithophilus pustulosus | PLPHI310-24 | BOLD:ACT8993 | PV175702 | PHILPL_0310 | Host: Calidris pugnax | N/A |
| Lunaceps holophaeus | PLPHI314-24 | BOLD:ACT8860 | PV175726 | PHILPL_0314 | Host: Calidris pugnax | GN36867 |
| Lunaceps holophaeus | PLPHI316-24 | BOLD:ACT8860 | PV175630 | PHILPL_0316 | Host: Calidris pugnax | GN36837 |
| Quadraceps obtusus | PLPHI319-24 | BOLD:ACT8857 | PV175602 | PHILPL_0319 | Host: Tringa totanus | GN36838 |
| Quadraceps obtusus | PLPHI322-24 | N/A | N/A | PHILPL_0322 | Host: Tringa totanus | GN27887 |
| Quadraceps furvus | PLPHI325-24 | N/A | N/A | PHILPL_0325 | Host: Tringa erythropus | GN21488 |
| Quadraceps furvus | PLPHI326-24 | BOLD:AFX5371 | PV175582 | PHILPL_0326 | Host: Tringa erythropus | GN36830 |
| Quadraceps furvus | PLPHI328-24 | BOLD:AFX5371 | PV175576 | PHILPL_0328 | Host: Tringa erythropus | GN36829 |
| Lunaceps falcinellus | PLPHI332-24 | BOLD:ACT8776 | PV175717 | PHILPL_0332 | Host: Calidris ferruginea | JT79698 |
| Lunaceps holophaeus | PLPHI333-24 | BOLD:ACT8860 | PV175634 | PHILPL_0333 | Host: Calidris ferruginea | JT79698 |
| Lunaceps falcinellus | PLPHI335-24 | BOLD:ACT8776 | PV175653 | PHILPL_0335 | Host: Calidris ferruginea | JT79705 |
| Lunaceps holophaeus | PLPHI337-24 | BOLD:ACT8860 | PV175675 | PHILPL_0337 | Host: Calidris ferruginea | JT79705 |
| Actornithophilus ochraceus | PLPHI338-24 | N/A | N/A | PHILPL_0338 | Host: Charadrius hiaticula | TS44182 |
| Quadraceps hiaticulae | PLPHI341-24 | BOLD:ACT8855 | PV175716 | PHILPL_0341 | Host: Charadrius hiaticula | TS44182 |
| Actornithophilus ochraceus | PLPHI342-24 | BOLD:ADA8181 | PV175649 | PHILPL_0342 | Host: Charadrius hiaticula | TS44183 |
| Quadraceps hiaticulae | PLPHI345-24 | BOLD:ACT8855 | PV175746 | PHILPL_0345 | Host: Charadrius hiaticula | TS44180 |
| Actornithophilus ochraceus | PLPHI346-24 | BOLD:ADA8181 | PV175617 | PHILPL_0346 | Host: Charadrius hiaticula | TS44179 |
| Actornithophilus ochraceus | PLPHI347-24 | BOLD:ADA8181 | PV175603 | PHILPL_0347 | Host: Charadrius hiaticula | TS44175 |
| Actornithophilus ochraceus | PLPHI348-24 | BOLD:ADA8181 | PV175642 | PHILPL_0348 | Host: Charadrius hiaticula | TS44171 |
| Actornithophilus ochraceus | PLPHI350-24 | BOLD:ADA8181 | PV175662 | PHILPL_0350 | Host: Charadrius hiaticula | TS44181 |
| Quadraceps hiaticulae | PLPHI352-24 | BOLD:ACT8855 | PV175745 | PHILPL_0352 | Host: Charadrius hiaticula | TS44178 |
| Actornithophilus ochraceus | PLPHI353-24 | BOLD:ADA8181 | PV175736 | PHILPL_0353 | Host: Charadrius hiaticula | TS44174 |
| Actornithophilus ochraceus | PLPHI355-24 | BOLD:ADA8181 | PV175733 | PHILPL_0355 | Host: Charadrius hiaticula | TS44184 |
| Actornithophilus ochraceus | PLPHI356-24 | N/A | N/A | PHILPL_0356 | Host: Charadrius hiaticula | JT79714 |
| Actornithophilus ochraceus | PLPHI360-24 | N/A | N/A | PHILPL_0360 | Host: Charadrius dubius | JT79694 |
| Actornithophilus ochraceus | PLPHI361-24 | BOLD:ADA8181 | PV175688 | PHILPL_0361 | Host: Charadrius dubius | JT79581 |
| Actornithophilus ochraceus | PLPHI363-24 | BOLD:ADA8181 | PV175656 | PHILPL_0363 | Host: Charadrius dubius | JT79693 |
| Actornithophilus ochraceus | PLPHI365-24 | BOLD:ADA8181 | PV175650 | PHILPL_0365 | Host: Charadrius dubius | JT79582 |
| Quadraceps bicuspis | PLPHI366-24 | BOLD:AGD1542 | PV175580 | PHILPL_0366 | Host: Charadrius dubius | JT79518 |
| Actornithophilus ochraceus | PLPHI367-24 | BOLD:ADA8181 | PV175681 | PHILPL_0367 | Host: Charadrius dubius | JT79631 |
| Quadraceps similis | PLPHI369-24 | BOLD:AES5355 | PV175616 | PHILPL_0369 | Host: Tringa nebularia | GN36834 |
| Actornithophilus paludosus | PLPHI372-24 | N/A | N/A | PHILPL_0372 | Host: Tringa nebularia | GN36832 |
| Quadraceps similis | PLPHI374-24 | BOLD:AES5355 | PV175668 | PHILPL_0374 | Host: Tringa nebularia | GN36832 |
| Quadraceps similis | PLPHI377-24 | BOLD:AES5355 | PV175723 | PHILPL_0377 | Host: Tringa nebularia | GN36847 |
| Quadraceps similis | PLPHI378-24 | BOLD:AES5355 | PV175686 | PHILPL_0378 | Host: Tringa nebularia | GN36828 |
| Actornithophilus paludosus | PLPHI381-24 | N/A | N/A | PHILPL_0381 | Host: Tringa nebularia | GN36830 |
| Quadraceps similis | PLPHI382-24 | BOLD:AES5355 | PV175629 | PHILPL_0382 | Host: Tringa nebularia | GN36839 |
| Actornithophilus paludosus | PLPHI384-24 | N/A | N/A | PHILPL_0384 | Host: Tringa nebularia | GN36839 |
| Lunaceps schismatus | PLPHI385-24 | BOLD:ACT8778 | PV175613 | PHILPL_0385 | Host: Calidris alpina | JT79709 |
| Lunaceps schismatus | PLPHI387-24 | BOLD:ACT8778 | PV175689 | PHILPL_0387 | Host: Calidris alpina | JT79721 |
| Lunaceps schismatus | PLPHI388-24 | N/A | N/A | PHILPL_0388 | Host: Calidris alpina | JT79719 |
| Lunaceps schismatus | PLPHI392-24 | N/A | N/A | PHILPL_0392 | Host: Calidris alpina | JT79708 |
| Lunaceps schismatus | PLPHI395-24 | BOLD:ACT8778 | PV175705 | PHILPL_0395 | Host: Calidris alpina | JT39720 |
| Lunaceps schismatus | PLPHI398-24 | BOLD:ACT8778 | PV175676 | PHILPL_0398 | Host: Calidris alpina | JT79710 |
| Lunaceps schismatus | PLPHI401-24 | BOLD:ACT8778 | PV175586 | PHILPL_0401 | Host: Calidris alpina | JT79700 |
| Lunaceps schismatus | PLPHI405-24 | BOLD:ACT8778 | PV175735 | PHILPL_0405 | Host: Calidris alpina | JT79615 |
| Lunaceps schismatus | PLPHI408-24 | N/A | N/A | PHILPL_0408 | Host: Calidris alpina | JT79707 |
| Lunaceps schismatus | PLPHI411-24 | N/A | N/A | PHILPL_0411 | Host: Calidris alpina | JT79716 |
| Lunaceps schismatus | PLPHI413-24 | N/A | N/A | PHILPL_0413 | Host: Calidris alpina | JT79717 |
| Lunaceps schismatus | PLPHI416-24 | N/A | N/A | PHILPL_0416 | Host: Calidris alpina | JT79712 |
| Lunaceps schismatus | PLPHI418-24 | BOLD:ACT8778 | PV175737 | PHILPL_0418 | Host: Calidris alpina | JT79711 |
| Lunaceps schismatus | PLPHI421-24 | BOLD:ACT8778 | PV175590 | PHILPL_0421 | Host: Calidris alpina | JT79713 |
| Lunaceps schismatus | PLPHI424-24 | N/A | N/A | PHILPL_0424 | Host: Calidris alpina | JT79699 |
| Lunaceps schismatus | PLPHI425-24 | BOLD:ACT8778 | PV175625 | PHILPL_0425 | Host: Calidris alpina | JT79706 |
| Lunaceps schismatus | PLPHI426-24 | BOLD:ACT8778 | PV175724 | PHILPL_0426 | Host: Calidris alpina | JT79718 |
| Lunaceps schismatus | PLPHI429-24 | BOLD:ACT8778 | PV175750 | PHILPL_0429 | Host: Calidris alpina | JT79558 |
| Carduiceps meinertzhageni | PLPHI430-24 | N/A | N/A | PHILPL_0430 | Host: Calidris alpina | JT79697 |
| Lunaceps schismatus | PLPHI433-24 | BOLD:ACT8778 | PV175729 | PHILPL_0433 | Host: Calidris alpina | JT80611 |
| Lunaceps schismatus | PLPHI434-24 | BOLD:ACT8778 | PV175584 | PHILPL_0434 | Host: Calidris alpina | JT80643 |
| Lunaceps schismatus | PLPHI436-24 | N/A | N/A | PHILPL_0436 | Host: Calidris alpina | JT35628 |
| Lunaceps schismatus | PLPHI438-24 | N/A | N/A | PHILPL_0438 | Host: Calidris alpina | JT80613 |
| Lunaceps schismatus | PLPHI439-24 | N/A | N/A | PHILPL_0439 | Host: Calidris alpina | JT80618 |
| Lunaceps schismatus | PLPHI442-24 | BOLD:ACT8778 | PV175697 | PHILPL_0442 | Host: Calidris alpina | JT80616 |
| Lunaceps schismatus | PLPHI445-24 | BOLD:ACT8778 | PV175643 | PHILPL_0445 | Host: Calidris alpina | JT80626 |
| Lunaceps schismatus | PLPHI448-24 | N/A | N/A | PHILPL_0448 | Host: Calidris alpina | JT80614 |
| Lunaceps schismatus | PLPHI451-24 | N/A | N/A | PHILPL_0451 | Host: Calidris alpina | JT80623 |
| Lunaceps schismatus | PLPHI454-24 | N/A | N/A | PHILPL_0454 | Host: Calidris alpina | JT80631 |
| Lunaceps schismatus | PLPHI457-24 | BOLD:ACT8778 | PV175637 | PHILPL_0457 | Host: Calidris alpina | JT80627 |
| Quadraceps hiaticulae | PLPHI460-24 | BOLD:ACT8855 | PV175612 | PHILPL_0460 | Host: Charadrius hiaticula | TS35301 |
| Lunaceps schismatus | PLPHI462-24 | BOLD:ACT8778 | PV175658 | PHILPL_0462 | Host: Calidris alpina | JT80615 |
| Lunaceps schismatus | PLPHI465-24 | BOLD:ACT8778 | PV175718 | PHILPL_0465 | Host: Calidris alpina | JT80604 |
| Lunaceps schismatus | PLPHI466-24 | N/A | N/A | PHILPL_0466 | Host: Calidris alpina | JT80629 |
| Lunaceps schismatus | PLPHI469-24 | N/A | N/A | PHILPL_0469 | Host: Calidris alpina | JT80607 |
| Lunaceps schismatus | PLPHI471-24 | N/A | N/A | PHILPL_0471 | Host: Calidris alpina | JT80620 |
| Lunaceps schismatus | PLPHI474-24 | BOLD:ACT8778 | PV175732 | PHILPL_0474 | Host: Calidris alpina | JT80608 |
| Lunaceps schismatus | PLPHI475-24 | N/A | N/A | PHILPL_0475 | Host: Calidris alpina | JT80608 |
| Lunaceps schismatus | PLPHI477-24 | N/A | N/A | PHILPL_0477 | Host: Calidris alpina | JT80630 |
| Lunaceps schismatus | PLPHI480-24 | N/A | N/A | PHILPL_0480 | Host: Calidris alpina | JT80609 |
| Lunaceps schismatus | PLPHI483-24 | BOLD:ACT8778 | PV175747 | PHILPL_0483 | Host: Calidris alpina | JT80610 |
| Lunaceps schismatus | PLPHI486-24 | N/A | N/A | PHILPL_0486 | Host: Calidris alpina | JT80625 |
| Lunaceps schismatus | PLPHI489-24 | BOLD:ACT8778 | PV175698 | PHILPL_0489 | Host: Calidris alpina | JT80644 |
| Lunaceps schismatus | PLPHI490-24 | BOLD:ACT8778 | PV175575 | PHILPL_0490 | Host: Calidris alpina | JT80642 |
| Lunaceps schismatus | PLPHI493-24 | BOLD:ACT8778 | PV175604 | PHILPL_0493 | Host: Calidris alpina | JT80645 |
| Lunaceps falcinellus | PLPHI495-24 | BOLD:ACT8776 | PV175640 | PHILPL_0495 | Host: Calidris minuta | NN03286 |
| Lunaceps falcinellus | PLPHI498-24 | BOLD:ACT8776 | PV175572 | PHILPL_0498 | Host: Calidris minuta | NN03285 |
| Lunaceps falcinellus | PLPHI499-24 | BOLD:ACT8776 | PV175722 | PHILPL_0499 | Host: Calidris temminckii | NN03282 |
| Lunaceps falcinellus | PLPHI500-24 | N/A | N/A | PHILPL_0500 | Host: Calidris temminckii | NN20714 |
| Lunaceps falcinellus | PLPHI502-24 | BOLD:ACT8776 | PV175693 | PHILPL_0502 | Host: Calidris minuta | NN20721 |
| Actornithophilus flavipes | PLPHI505-24 | BOLD:ACT8711 | PV175614 | PHILPL_0505 | Host: Pluvialis squatarola | GN36835 |
| Cummingsiella ovalis | PLPHI507-24 | BOLD:ADA6787 | PV175609 | PHILPL_0507 | Host: Numenius arquata | ES09791 |
| Lunaceps numenii | PLPHI508-24 | BOLD:ACT8858 | PV175657 | PHILPL_0508 | Host: Numenius arquata | ES09791 |
| Saemundssonia platygaster | PLPHI511-24 | BOLD:AGC6064 | PV175710 | PHILPL_0511 | Host: Actitis hypoleucos | JT25390 |

**Table S2.** Models assessing variation in chewing louse infestation traits (prevalence and abundance) among host species, host age classes, sites, and years in shorebirds migrating through central Poland. Significant effects are indicated in bold.

| Infestation trait | Predictor | β±SE | F value | *P* | Partial R^2^ |
| --- | --- | --- | --- | --- | --- |
| Prevalence | **Host species** | **-** | **24.53** | **< 0.001** | **-** |
|  | **Host age class _(first-year)_** | **-1.37±0.29** | **24.83** | **< 0.001** | **0.013** |
|  | **Site _(river valley)_** | **-1.11±0.2** | **21.11** | **< 0.001** | **0.013** |
|  | **Year** | **-** | **39.08** | **< 0.001** | **-** |
| Abundance | **Host species** | **55.56** | **55.56** | **< 0.001** | **-** |
|  | **Host age class _(first-year)_** | **-1.40±0.01** | **18.73** | **< 0.001** | **0.006** |
|  | **Site _(river valley)_** | **-0.31±0.06** | **4.27** | **0.040** | **0.001** |
|  | **Year** | **-** | **103.88** | **< 0.001** | **-** |

**Table S3.** Models assessing relationships between chewing louse abundance and host body condition (fat load and body mass) in first-year wood sandpipers and common snipes migrating through central Poland. Significant effects are indicated in bold.

| Species | Host body condition | Predictor | β ± SE | *Z* | *P* |
| --- | --- | --- | --- | --- | --- |
| Wood sandpiper | Fat load | **Intercept** | **6.217 ± 0.712** | **8.730** | **< 0.001** |
|  |  | **Fat load** | **-0.105 ± 0.024** | **-4.385** | **< 0.001** |
|  |  | **Wing length** | **-0.029 ± 0.005** | **-5.322** | **< 0.001** |
|  |  | **Day** | **0.006 ± 0.002** | **2.595** | **0.009** |
|  |  | **Year 2020** | **-1.288 ± 0.050** | **-25.627** | **< 0.001** |
|  |  | **Year 2021** | **-1.688 ± 0.067** | **-25.292** | **< 0.001** |
|  | Body mass | **Intercept** | **6.103 ± 0.710** | **8.598** | **< 0.001** |
|  |  | **Body mass** | **-0.012 ± 0.002** | **-4.738** | **< 0.001** |
|  |  | **Wing length** | **-0.024 ± 0.005** | **-4.211** | **< 0.001** |
|  |  | **Day** | **0.006 ± 0.002** | **2.554** | **0.011** |
|  |  | **Year 2020** | **-1.283 ± 0.050** | **-25.757** | **< 0.001** |
|  |  | **Year 2021** | **-1.701 ± 0.067** | **-25.274** | **< 0.001** |
| Common snipe | Fat load | **Intercept** | **6.881 ± 3.116** | **2.208** | **0.027** |
|  |  | Fat load | -0.008 ± 0.098 | -0.084 | 0.933 |
|  |  | **Wing length** | **-0.043 ± 0.022** | **-1.922** | **0.055** |
|  |  | **Day** | **-0.023 ± 0.009** | **-2.514** | **0.012** |
|  |  | **Year 2020** | **-0.910 ± 0.176** | **-5.163** | **< 0.001** |
|  |  | **Year 2021** | **-1.445 ± 0.213** | **-6.788** | **< 0.001** |
|  | Body mass | **Intercept** | **6.827 ± 3.125** | **2.184** | **0.029** |
|  |  | Body mass | 0.001 ± 0.006 | 0.229 | 0.819 |
|  |  | **Wing length** | **-0.044 ± 0.023** | **-1.943** | **0.052** |
|  |  | **Day** | **-0.023 ± 0.009** | **-2.512** | **0.012** |
|  |  | **Year 2020** | **-0.899 ± 0.171** | **-5.261** | **< 0.001** |
|  |  | **Year 2021** | **-1.442 ± 0.210** | **-6.854** | **< 0.001** |

**Table S4.** Models assessing relationships between chewing louse prevalence and host body condition (fat load and body mass) in first-year wood sandpipers and common snipes migrating through central Poland. Significant effects are indicated in bold.

| Species | Host body condition | Predictor | β ± SE | *Z* | *P* |
| --- | --- | --- | --- | --- | --- |
| Wood sandpiper | Fat load | **Intercept** | **5.911 ± 4.067** | **1.454** | **0.146** |
|  |  | Fat load | -0.075 ± 0.129 | -0.584 | 0.559 |
|  |  | Wing length | -0.025 ± 0.031 | -0.813 | 0.416 |
|  |  | Day | -0.019 ± 0.016 | -1.203 | 0.229 |
|  |  | **Year 2020** | **-0.757 ± 0.313** | **-2.415** | **0.016** |
|  |  | **Year 2021** | **-2.255 ± 0.363** | **-6.206** | **<0.001** |
|  | Body mass | Intercept | 5.799 ± 4.069 | 1.425 | 0.154 |
|  |  | Body mass | -0.011 ± 0.014 | -0.767 | 0.443 |
|  |  | Wing length | -0.020 ± 0.032 | -0.643 | 0.520 |
|  |  | Day | -0.017 ± 0.016 | -1.059 | 0.289 |
|  |  | **Year 2020** | **-0.765 ± 0.313** | **-2.445** | **0.015** |
|  |  | **Year 2021** | **-2.254 ± 0.359** | **-6.283** | **<0.001** |
| Common snipe | Fat load | Intercept | 8.099 ± 5.624 | 1.440 | 0.149 |
|  |  | Fat load | 0.100 ± 0.187 | 0.537 | 0.591 |
|  |  | Wing length | -0.049 ± 0.040 | -1.220 | 0.222 |
|  |  | **Day** | **-0.048 ± 0.019** | **-2.593** | **0.009** |
|  |  | **Year 2020** | **-0.752 ± 0.326** | **-2.305** | **0.021** |
|  |  | **Year 2021** | **-1.501 ± 0.376** | **-3.997** | **< 0.001** |
|  | Body mass | Intercept | 7.810 ± 5.618 | 1.390 | 0.164 |
|  |  | Body mass | 0.002 ± 0.013 | 0.197 | 0.844 |
|  |  | Wing length | -0.048 ± 0.040 | -1.191 | 0.234 |
|  |  | **Day** | **-0.048 ± 0.018** | **-2.581** | **0.009** |
|  |  | **Year 2020** | **-0.798 ± 0.313** | **-2.548** | **0.011** |
|  |  | **Year 2021** | **-1.535 ± 0.369** | **-4.155** | **< 0.001** |
